# Autophagy-Related Three-Gene Prognostic Signature for Predicting Survival in Esophageal Squamous Cell Carcinoma

**DOI:** 10.3389/fonc.2021.650891

**Published:** 2021-07-15

**Authors:** Heyang Cui, Yongjia Weng, Ning Ding, Chen Cheng, Longlong Wang, Yong Zhou, Ling Zhang, Yongping Cui, Weimin Zhang

**Affiliations:** ^1^Department of Oncology, Cancer Institute, Peking University Shenzhen Hospital, Shenzhen Peking University-Hong Kong University of Science and Technology (PKU-HKUST) Medical Center, Shenzhen, China; ^2^Key Laboratory of Carcinogenesis and Translational Research (Ministry of Education/Beijing), Laboratory of Molecular Oncology, Peking University Cancer Hospital & Institute, Beijing, China

**Keywords:** ESCC, autophagy, PARP1, ITGA6, FADD, prognosis

## Abstract

Esophageal squamous cell carcinoma (ESCC) is one of the most aggressive malignant tumors in China, and its prognosis remains poor. Autophagy is an evolutionarily conserved catabolic process involved in the occurrence and development of ESCC. In this study, we described the expression profile of autophagy-related genes (ARGs) in ESCC and developed a prognostic prediction model for ESCC patients based on the expression pattern of ARGs. We used four ESCC cohorts, GSE53624 (119 samples) set as the discovery cohort, The Cancer Genome Atlas (TCGA) ESCC set (95 samples) as the validation cohort, 155 ESCC cohort, and Oncomine cohort were used to screen and verify differentially expressed ARGs. We identified 34 differentially expressed genes out of 222 ARGs. In the discovery cohort, we divided ESCC patients into three groups that showed significant differences in prognosis. Then, we analyzed the prognosis of 34 differentially expressed ARGs. Three genes [poly (ADP-ribose) polymerase 1 (PARP1), integrin alpha-6 (ITGA6), and Fas-associated death domain (FADD)] were ultimately obtained through random forest feature selection and were constructed as an ARG-related prognostic model. This model was further validated in TCGA ESCC set. Cox regression analysis confirmed that the three-gene signature was an independent prognostic factor for ESCC patients. This signature effectively stratified patients in both discovery and validation cohorts by overall survival (*P* = 5.162E-8 and *P* = 0.052, respectively). We also constructed a clinical nomogram with a concordance index of 0.713 to predict the survival possibility of ESCC patients by integrating clinical characteristics and the ARG signature. The calibration curves substantiated fine concordance between nomogram prediction and actual observation. In conclusion, we constructed a new ARG-related prognostic model, which shows the potential to improve the ability of individualized prognosis prediction in ESCC.

## Introduction

Esophageal cancer is one of the most common malignant tumors of the digestive system, with high morbidity and mortality (1). It has two major histological types: esophageal adenocarcinoma (EAC) and esophageal squamous cell carcinoma (ESCC) (2). ESCC is the principal histological type in China, which has the highest incidence and mortality compared with other countries (3). Despite the technical developments in diagnosis and treatment, this disease still tends to have a poor prognosis (2, 4) due to late diagnosis and lack of effective targets. Better understanding of the genetic and molecular disorders of the disease is the key to early diagnosis, appropriate treatment, and improved prognosis of patients with ESCC.

Autophagy is a critical and intricate homeostatic process in cells that is involved in a variety of biological processes (5). When exposed to various external stimuli, such as starvation, hypoxia, and drug, the magnitude of autophagy may increase sharply to provide nutrients and remove harmful substances (6). It suggests that autophagy is subjected to highly orchestrated regulation, including phosphoinositide 3-kinase (PI3K)/AKT/mammalian target of rapamycin (mTOR), p53/damage-regulated autophagy modulator (DRAM), Janus kinase (JAK)–signal transducer and activator of transcription (STAT), RAS, and AMP-activated protein kinase (AMPK)/calcium/calmodulin-dependent protein kinase kinase (CaMKK) signaling pathways; some known signaling pathways regulating critical cell cycle are all related to autophagy (7).

Autophagy is generally regarded as a double-edged sword in tumors (8). It may have the opposite effect depending on the tumor type, clinical stage, genetic background, or treatment, which either suppresses or promotes tumor development (9). In general, autophagy can prevent carcinogenesis by removing carcinogenic protein substrates, misfolded proteins, and damaged organelles (8). However, in established cancer, autophagy can meet the needs of tumor growth by recycling macromolecules and organelles (10). At present, autophagy has been gradually used in the diagnosis and treatment of tumors in some studies. Its inhibitor chloroquine and hydroxychloroquine have been used in clinical treatment (7). These drugs alone or in combination have been used in clinical trials of some tumors, including melanoma, colorectal cancer, myeloma, and renal cell carcinoma. The results show that autophagy inhibitors have certain therapeutic potential (11–13). However, although autophagy has been found to be associated with chemotherapy resistance in esophageal squamous cell lines (14, 15), its roles and clinical value have not been tested in patients with ESCC. Thus, it is of great significance to find suitable molecular biomarkers with autophagy as the core for prognosis prediction and treatment of ESCC.

In this study, we aimed to explore autophagy-related genes (ARGs) involved in ESCC progression. Gene expression data from public databases Gene Expression Omnibus (GEO) were used to classify subtypes of ESCC and established prognosis risk model based on ARGs. The relationships between the molecular subtypes and prognosis and clinical characteristics of ESCC patients were further evaluated. The three-gene prognostic risk model constructed with the differentially expressed ARGs among ESCC can better evaluate the prognosis of ESCC samples. Furthermore, TCGA gene expression data set was used to further verify the well-performance of the prognostic risk model.

## Materials and Methods

### Selection of Autophagy-Related Genes

The 222 ARGs were collected from Human Autophagy Database (HADb; http://www.autophagy.lu/clustering/) in March 2019. And 222 ARGs were listed in Supplementary Table 1.

### Data Acquisition and Processing

The expression data of the GSE53624 dataset and clinical characteristics of ESCC cohorts were obtained from the GEO (https://www.ncbi.nlm.nih.gov/geo/query/acc.cgi?acc=GSE53624). The expression data of TCGA ESCC RNA sequencing (RNA-seq) dataset were downloaded from TCGA website (https://portal.gdc.cancer.gov/) for the validation studies. We used our own ESCC cohort containing 155 ESCC RNA-seq data to screen the differentially expressed ARGs. In this cohort, we performed RNA-seq on fresh tumor specimens and matched adjacent normal tissues from 155 ESCC patients recruited from Shanxi province, China. In addition, we also verified the differentially expressed genes (DEGs) in 53 pairs of ESCC from the Oncomine database (https://www.oncomine.org).

### RNA Sequencing and Gene Expression Analysis

Total RNA was extracted from frozen samples using the TRIzol reagent (Life Technologies, Carlsbad, CA, USA), and DNA was digested by DNase I following the instructions of the manufacturer. RNA quantity and quality were evaluated by NanoDrop spectrophotometer (Thermo Scientific, USA). Here, 1% gel electrophoresis was used to determine the RNA integrity. Enriched mRNA with Oligo (dT) were broken into fragments for the preparation of cDNA libraries. The cDNA libraries were quality inspection qualified with the Agilent 2100 Bioanalyzer and ABI Step One Plus Real-Time PCR System, then sequenced on Illumina HiSeq X Ten.

Over 50M raw reads were sequenced for each sample. Raw reads were trimmed by Skewer (v0.2.2) (16) to remove adapter sequences and then aligned against reference genome (GRCh37/hg19) by STAR (v2.4.2a) (17). RSEM (1.2.29) (18) was used to perform expression abundance quantification based on the uniquely mapped reads. Gene annotation GENCODE v19 was used in the above process.

### Screening of Differentially Expressed Autophagy-Related Genes

Student's *t*-test, receiver operating characteristic (ROC), and GEO2R (https://www.ncbi.nlm.nih.gov/geo/geo2r) were used to screen the DEGs between ESCC and normal tissue. Genes with area under the ROC curve (AUC) ≥0.85, *q* < 0.0001, |log2(FC)| ≥ 0.5 were selected as the significantly differentially expressed ARGs.

#### First-Round Validation

We used the 155-ESCC dataset to verify the differentially expressed identified ARGs. EdgeR package in R statistical software was applied to estimate differentially expressed ARGs between ESCC and normal samples (*q* < 0.0001 and log2(FC) ≥0.5 or ≤ -0.5).

#### Second-Round Validation

The first-round validated ARGs were further verified using the Oncomine database (https://www.oncomine.org/resource/main.html). Very strict thresholds were applied, *P* ≤ 0.0001, log2(FC) ≥0.5 or ≤-0.5.

### Cluster to Identify Subtypes

We used 34 identified differentially ARGs to cluster analysis. Ward.D2 algorithm was used to cluster the ESCC samples. And then we used pheatmap of R to draw cluster heatmap, annotated by clinical features, including Age, Stage, Lymph node metastasis, Location, Drinking, Smoking, and Gender.

### Construction of a Prognostic Gene Signature Based on Autophagy-Related Genes

Univariate Cox regression analyses were performed to select the ARGs whose expression profiles were significantly associated with ESCC patient's overall survival (OS) (*P* < 0.1). And then we further used the random survival forest algorithm to rank the importance of prognostic ARGs. R package random survival forest was used to screen the prognostic genes. We set the number of Monte Carlo iterations to 100 and the number of steps forward to 5 and identified the genes whose relative importance as characteristic genes was >0.3. Finally, we carried out a multivariate Cox regression analysis and constructed a risk scoring model:

$$\text{Risk Score}=\sum_{k-1}^{n}\left(Exp_{k}*\;e_{K}^{HR}\right)$$

N is the number of prognostic ARGs, *Exp*_*k*_ is the expression value of the ARGs, and $e_{k}^{HR}$ is the estimated regression coefficient of genes in the multivariate Cox regression analysis.

### Functional Enrichment Analysis

We performed a series of gene functional enrichment analyses with DEGs, including Gene Ontology (GO) and Kyoto Encyclopedia of Genes and Genomes (KEGG). The Database for Annotation, Visualization, and Integrated Discovery [DAVID (19); https://david.ncifcrf.gov/] was used to identify enriched GO and KEGG terms. And we used GOplot package of R to visualize the results of enrichment analysis.

### Development of the Nomogram

Significant factors of univariate analysis (Age, Stage, and Risk score) were used to construct a nomogram by the survival and the rms package for R. And we used the concordance index (C-index) to assess the model performance for predicting prognosis. Following that, calibration curves were plotted to evaluate the concordance between actual and predicted survival.

### Statistical Analysis

All statistics were executed using the R software (Version 4.0.2; https://www.R-project.org) and SPSS software (Version 22.0; https://www.ibm.com/analytics/spss-statistics-software). Student's *t*-test was used to compare the expression between tumor and normal samples. Fisher exact test was used to check the association of risk scores with clinical characteristics. Kaplan–Meier (KM) curves were plotted and a log-rank test and univariate Cox proportional hazard regression analysis were used to check the significant difference in OS. Univariate and multivariate Cox proportional hazard regression analysis was also performed to assess the association between risk score or clinical characteristics and OS. The ROC analysis was used to examine the sensitivity and specificity. An AUC served as an indicator of prognostic accuracy. A *P* < 0.05 or 0.1 was set as statistically significant.

## Results

### Differentially Expressed Autophagy-Related Genes in Esophageal Squamous Cell Carcinoma

**Figure 1:**
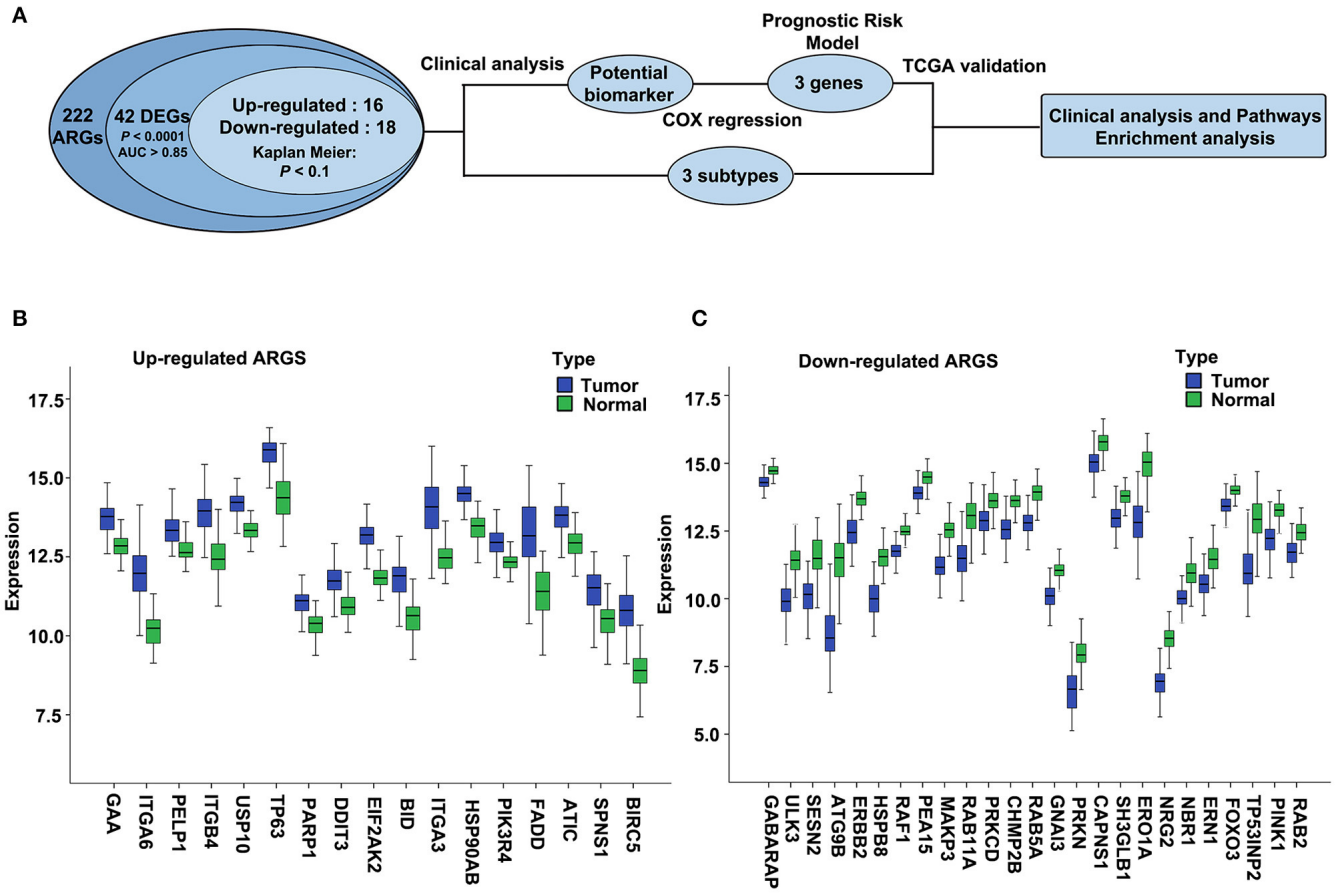
The overall flowchart and significantly differentially expressed autophagy-related genes (ARGs) in esophageal squamous cell carcinoma (ESCC). **(A)** Procedure for the selection and validation of the prognostic risk model in ESCC. DEGs, differentially expressed genes. **(B,C)** The expression patterns of 42 ARGs in ESCC and paired normal samples. Blue box represents tumor, and green box represents normal. **(B)** Upregulated ARGs. **(C)** Downregulated ARGs.

We used ESCC dataset GSE53624, which contains 222 ARGs (Supplementary Table 1, collected from HADb) from 119 ESCC and paired normal esophageal tissues to determine the differentially expressed ARGs. The overall flowchart of this study is shown in Figure 1A. Expression of 42 ARGs was found to more effectively discriminate ESCC from normal esophagus with AUC ≥ 0.85, *q* < 0.0001, |log2(FC)| ≥ 0.5 (Supplementary Table 2), including 17 upregulated ARGs (Figure 1B) and 25 downregulated ARGs (Figure 1C).

### Analysis and Validation of the Differentially Expressed Autophagy-Related Genes in the 155 Esophageal Squamous Cell Carcinoma Dataset and Oncomine Dataset

We then validated the 42 differentially expressed ARGs in an ESCC RNA-seq dataset, which contains 155 ESCC and paired normal esophagus. Here, 34 overlapping ARGs showed the significant differential expression with *q* < 0.0001 and log2(FC) ≥0.5 or ≤-0.5 (Supplementary Table 3), and the trend of ARG differential expression in the two groups was consistent, including 16 upregulated genes [EIF2AK2, BIRC5, HSP90AB1, BID, integrin alpha-6 (ITGA6), GAA, TP63, ITGB4, poly (ADP-ribose) polymerase 1 (PARP1), ITGA3, ATIC, Fas-associated death domain (FADD), PELP1, DDIT3, PIK3R4, SPNS1] and 18 downregulated genes (CAPNS1, FOXO3, SESN2, PARK2, GNAI3, SH3GLB1, ATG9B, ULK3, PINK1, ERO1L, CHMP2B, TP53INP2, RAB11A, NRG2, ERBB2, MAPK3, RAB5A, and HSPB8). We further verified the differential expression trend of these genes in ESCCs of the Oncomine database (Supplementary Table 3).

### Functional Annotation of the 34 Differentially Expressed Autophagy-Related Genes

Functional enrichment analysis of the 34 differentially expressed ARGs offered the biological understanding of these genes. According to the results of DAVID, the top enriched GO terms for cellular components were cytosol, membrane, mitochondrion, protein complex, cytoplasmic vesicle, integrin complex, late endosome, extracellular exosome, cell–cell adherens junction, cytoplasm, and autophagosome. For the molecular function, genes were mostly enriched in terms of protein binding, identical protein binding, and cadherin binding involved in cell–cell adhesion (Supplementary Figure 1). KEGG pathways enrichment analysis for the 34 differentially expressed ARGs showed that these genes were notably associated with pathways in cancer, focal adhesion, and PI3K–AKT signaling pathway (Supplementary Figure 2A). The heatmap of the relationship between ARGs and pathways was also displayed (Supplementary Figure 2B), including the focal adhesion and PI3K–AKT signaling pathway, which is consistent with previous studies (20–27).

### Molecular Typing Based on Autophagy-Related Genes

**Figure 2:**
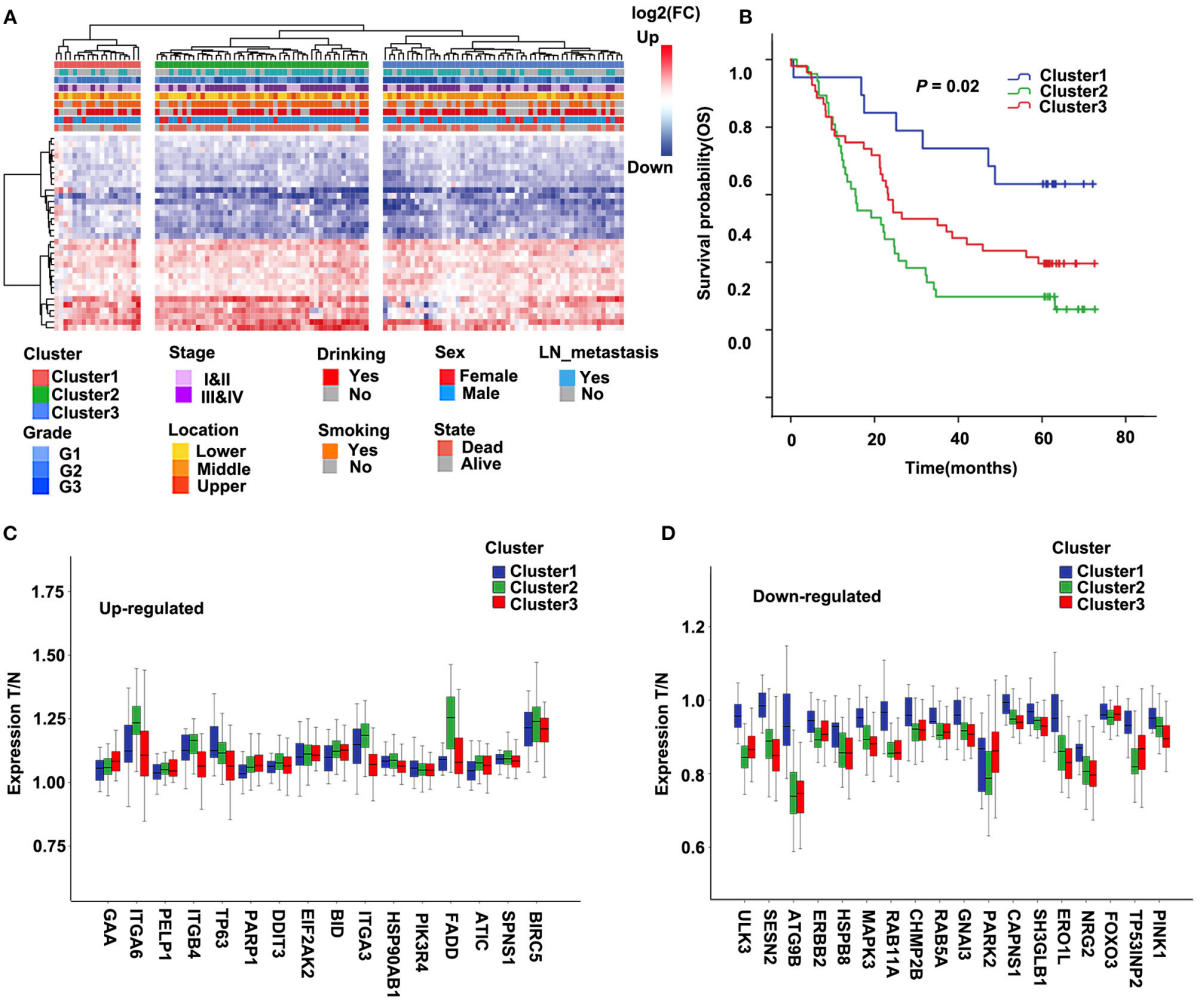
Cluster of differentially expressed autophagy-related genes (ARGs) and Kaplan–Meier (KM) survival plot in the discovery cohort (GSE53624). **(A)** The heatmap of 34 ARGs. Left: Annotation of cluster and clinical features. The color represents logFC of differential expression. LN, lymph node. **(B)** Survival curve and Kaplan–Meier analysis of esophageal squamous cell carcinoma (ESCC) patients by the cluster. OS, overall survival. **(C,D)** The ratio of tumor to normal expression of up- and down-ARGs in Cluster1–3; the ordinate shows the ratio of tumor to normal after normalization of expression.

Molecular subtypes were identified using the cluster method Ward.D2 based on 34 selected differentially expressed ARGs, and the optimal clustering number of 3 was selected (Figure 2A). We analyzed the prognosis of these three groups. The results showed that Cluster1 had a relatively better survival followed by Cluster3, whereas Cluster2 had the worst prognosis (Figure 2B; *P* = 0.02). The relationships between the subtypes and clinicopathological parameters (Age, Gender, Smoking, Drinking, Location, Grade, Stage, and Lymph node metastasis) of ESCC patients were summarized in Supplementary Table 4. We observed significant correlations between subtypes and Drinking (*P* = 0.01), Location (*P* = 0.092), Grade (*P* = 0.009), Stage (*P* = 0.01), and Lymph node metastasis (*P* = 0.076). Compared to Cluster1, patients in Cluster2 and Cluster3 had higher grade and stage. In Cluster3, the proportion of patients with alcohol drinking was relatively higher. The tumor location of Cluster1 and Cluster3 was more in the middle, while Cluster2 was more in the middle and lower sections of the esophagus.

Further, we compared the differentially expressed ARGs among these three groups. For most genes of 34 ARGs, the degree of upregulated ARGs in Cluster2 was significantly higher than the other two groups, and the degree of downregulated ARGs in Cluster2 and Cluster3 was significantly higher than Cluster1 (Figures 2C,D). This result may suggest that the changes of autophagy activities are related to the prognosis of ESCC patients.

### Construction of a Prognostic Risk Model Based on These 34 Autophagy-Related Genes

**Figure 3:**
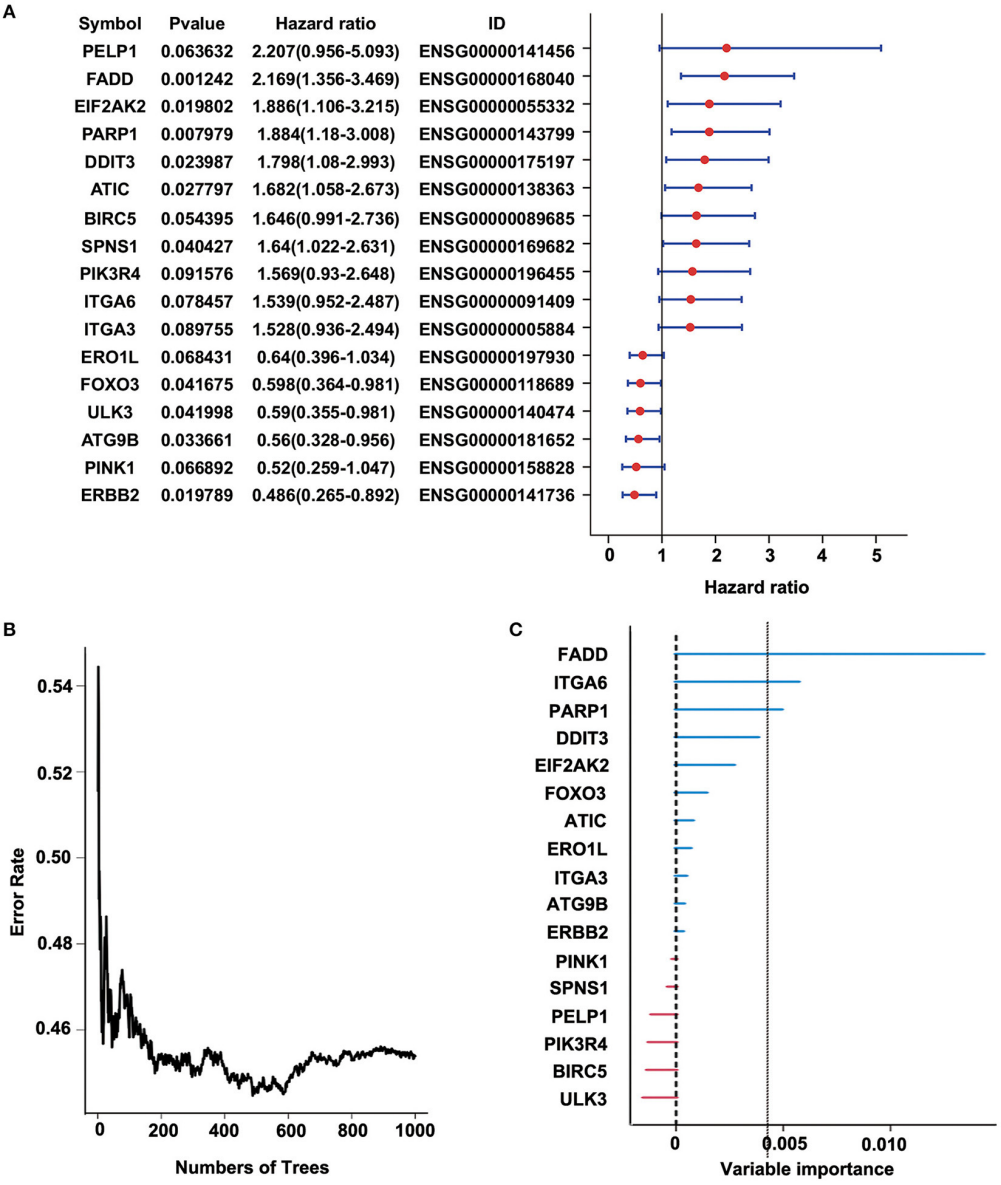
Random forest analysis of prognosis-related autophagy-related genes (ARGs) in the discovery cohort (GSE53624). **(A)** Forest plot of ARGs with esophageal squamous cell carcinoma (ESCC) survival, univariate Cox regression. **(B)** Relationship between the error rate and the number of classification trees. **(C)** Out-of-bag importance values for the predictors.

To identify a prognostic risk model, we analyzed the relationship between the expression of 34 ARGs and the prognosis of ESCC patients in the discovery cohort GSE53624 and selected 17 ARGs with significant *P*-value of univariate Cox regression (Figure 3A) as candidate genes. We used random forests for feature selection. The relationship between error rate and number of taxonomic trees was used to reveal genes with relative importance >0.3 as the final model (Figures 3B,C). We identified three genes, FADD, PARP1, and ITGA6 in this model (Table 1, Supplementary Figure 3). The important order of the out-of-bag scores for the three genes is displayed in Figure 3C. A three-gene prognostic risk model was established by multivariate COX regression analysis. The equation is as follows:

$$\begin{array}{l} \text{Risk Score}=0.184\;*\text{ expFADD}+0.562\;*\;\exp\text{PARP}1\\ \;+0.199\;*\;\exp\text{ITGA}6 \end{array}$$

**Table 1:** Three genes significantly associated with overall survival in the discovery cohort (GSE53624).

	**Symbol**	**HR**	***P***	**Importance**	**Relative imp**
ENSG00000168040	FADD	2.169	0.001242	0.0143	1
ENSG00000091409	ITGA6	1.539	0.078457	0.0057	0.3987
ENSG00000143799	PARP1	1.884	0.007979	0.0049	0.3438

*FADD, Fas-associated death domain; HR, hazard ratio; Imp, importance; ITGA6, integrin alpha-6; PARP1, poly (ADP-ribose) polymerase 1*.

**Figure 4:**
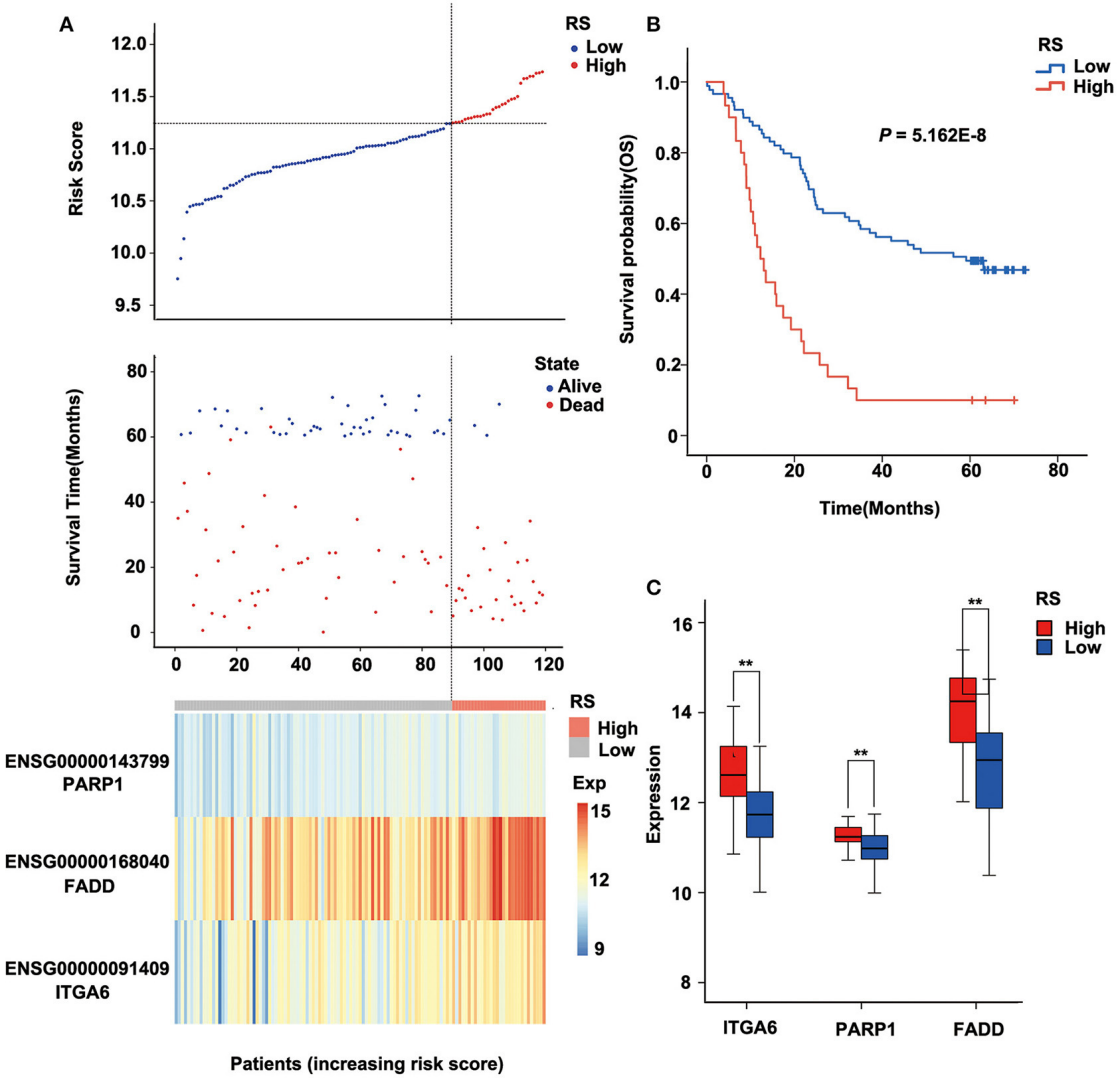
Relation between the three-gene signature and cancer risk in the discovery cohort (GSE53624). **(A)** Risk score, survival time, survival state, and expression of the three ARGs in esophageal squamous cell carcinoma (ESCC) patients of GSE53624. **(B)** Kaplan–Meier analysis of ESCC patients grouped by risk score; log-rank Mantel–Cox test was used to compare survival curves. **(C)** Box plot of expression of three ARGs grouped by risk score, and the independent-sample Student t-test was used as the comparison method between the high- and low-risk groups. RS, risk score; Exp, expression. **P < 0.01.

The risk score of each sample was calculated, ROC curve was constructed according to the value of risk and survival of patients, and the samples were divided into high-risk group and low-risk group with the maximum of Youden index. The prognosis of the high-risk and low-risk groups were significantly different (*P* = 5.162E-8; Figure 4B). High expression of FADD, PARP1, and ITGA6 was associated with high risk (Figures 4A,C).

Furthermore, we analyzed the prognosis of risk groups and their relationship with cluster, which was constructed by differentially expressed ARGs (Supplementary Table 5). The results show that high-risk patients were enriched in Cluster2 and Cluster3, and the high-risk patients accounted for the highest proportion in Cluster2, which had the worst prognosis (*P* = 6.511E-07; Supplementary Table 5, Supplementary Figure 4). There was no correlation between other clinical factors and risk groups (Supplementary Table 5).

### Verification of the Robustness of the Three-Gene Signature Model in The Cancer Genome Atlas Esophageal Squamous Cell Carcinoma Dataset

**Figure 5:**
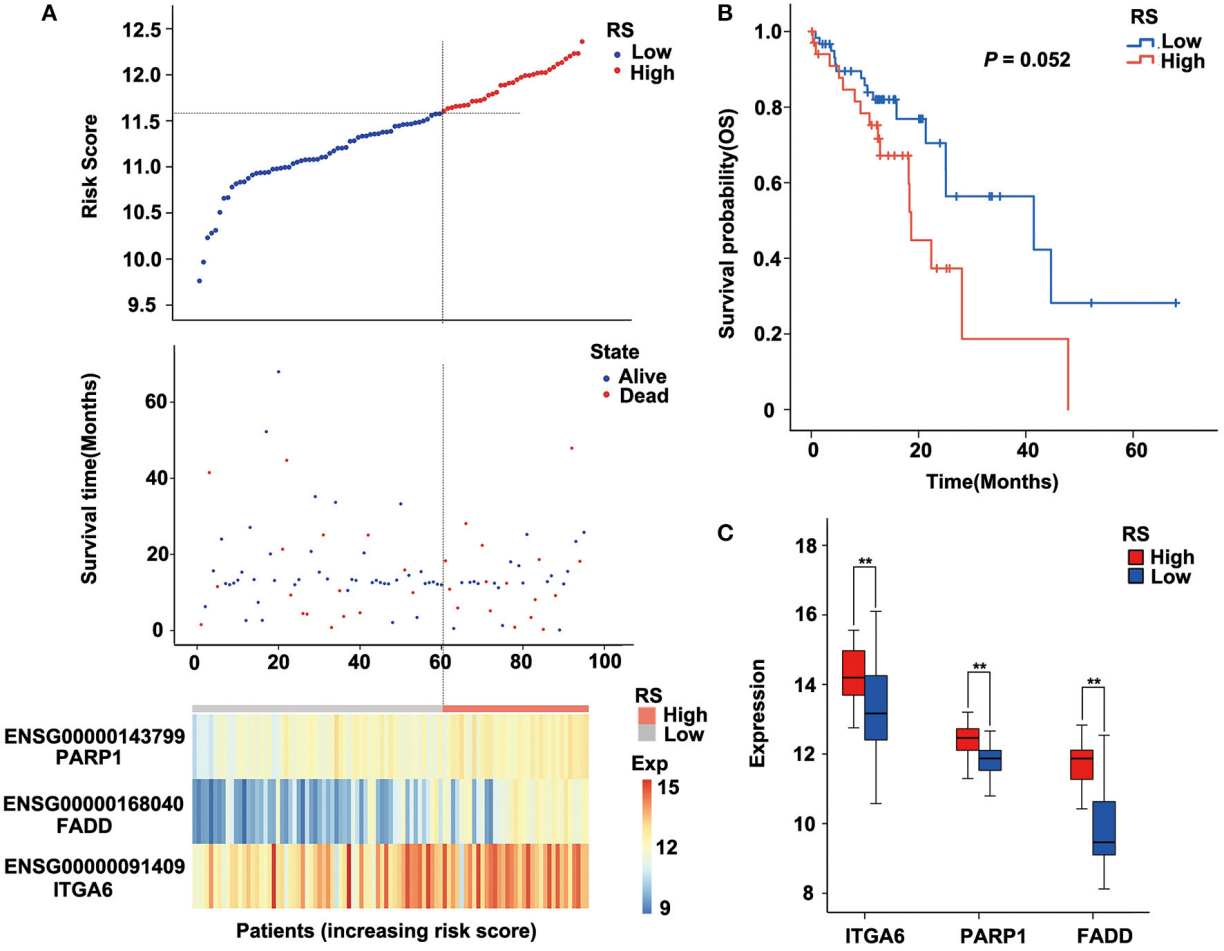
Performance of the three-gene signature model in the validation cohort [The Cancer Genome Atlas (TCGA) data]. **(A)** Risk score, survival time, survival state, and expression of the three ARGs in esophageal squamous cell carcinoma (ESCC) patients of TCGA. **(B)** Kaplan–Meier analysis of ESCC patients grouped by risk score; log-rank Mantel–Cox test was used to compare survival curves. **(C)** Box plot of expression of three ARGs grouped by risk score, and the independent-sample Student *t*-test was used as the comparison method between the high- and low-risk groups. RS, risk score; Exp, expression. **P < 0.01.

To verify the robustness of the three-gene signature model, we calculated a risk score for each sample in another validation cohort TCGA ESCC dataset. We used the same method to divide the 95 samples into high-risk and low-risk groups in discovery cohort GSE53624. The prognosis of the low-risk group was significantly better than that of the high-risk group (*P* = 0.052; Figure 5B, Supplementary Table 6). As shown in Figure 5, TCGA data revealed that the relationship between the expression of the three genes and risk score is also consistent with the GSE53624. Thus, the three-gene signature model we constructed was effective to predict prognosis for ESCC patients.

### Risk Model and Clinical Characteristic Analysis

**Figure 6:**
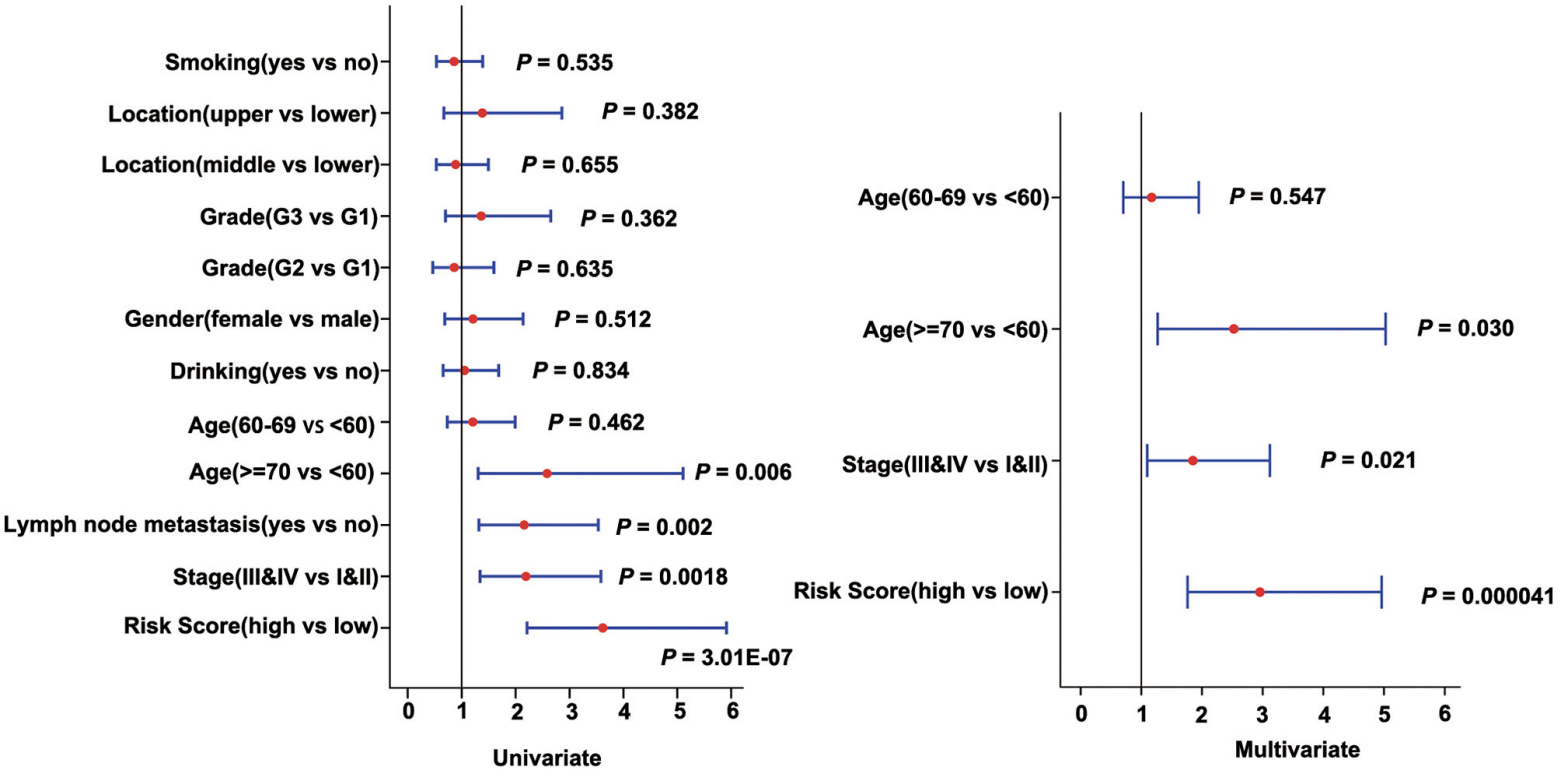
Forest plot of univariate and multivariate Cox regression analyses in the discovery cohort (GSE53624).

To assess the independence of the three-gene signature model in clinical application, we used univariate and multivariate Cox regression to analyze hazard ratio (HR), 95% confidence interval (CI), and *P*-values. We systematically analyzed the clinical information from the patients as recorded in ESCC, including their Age, Gender, Location, Smoking, Drinking, Grade, Stage, as well as our three-gene signature (Figure 6, Supplementary Table 7). In ESCC, univariate Cox regression analysis revealed that the risk score group (HR = 3.617, 95% CI = 2.212–5.914, *P* = 3.008E-07), Age (*P* = 0.024), Stage (HR = 2.19, 95% CI = 1.339–3.582, *P* = 0.002), and Lymph node metastasis (HR = 2.159, 95% CI = 1.319–3.534, *P* = 0.002) had clinical independence. And the corresponding multivariate Cox regression analysis found that the Risk score group (HR = 2.955, 95% CI = 1.761–4.961, *P* = 4.100E-05), Age (*P* = 0.03), and Stage (HR = 1.849, 95% CI = 1.096–3.12, *P* = 0.021) had clinical independence. Importantly, the validation data (TCGA cohort) also confirmed these findings (HR = 1.971, 95% CI = 0.982–3.955, *P* = 0.056 for univariate Cox regression analysis; HR = 2.67, 95% CI = 1.25–5.704, *P* = 0.011 for multivariate Cox regression analysis; Supplementary Table 8, Supplementary Figure 5A), suggesting that our three-gene signature model may serve as an independent prognostic index for clinical application.

**Figure 7:**
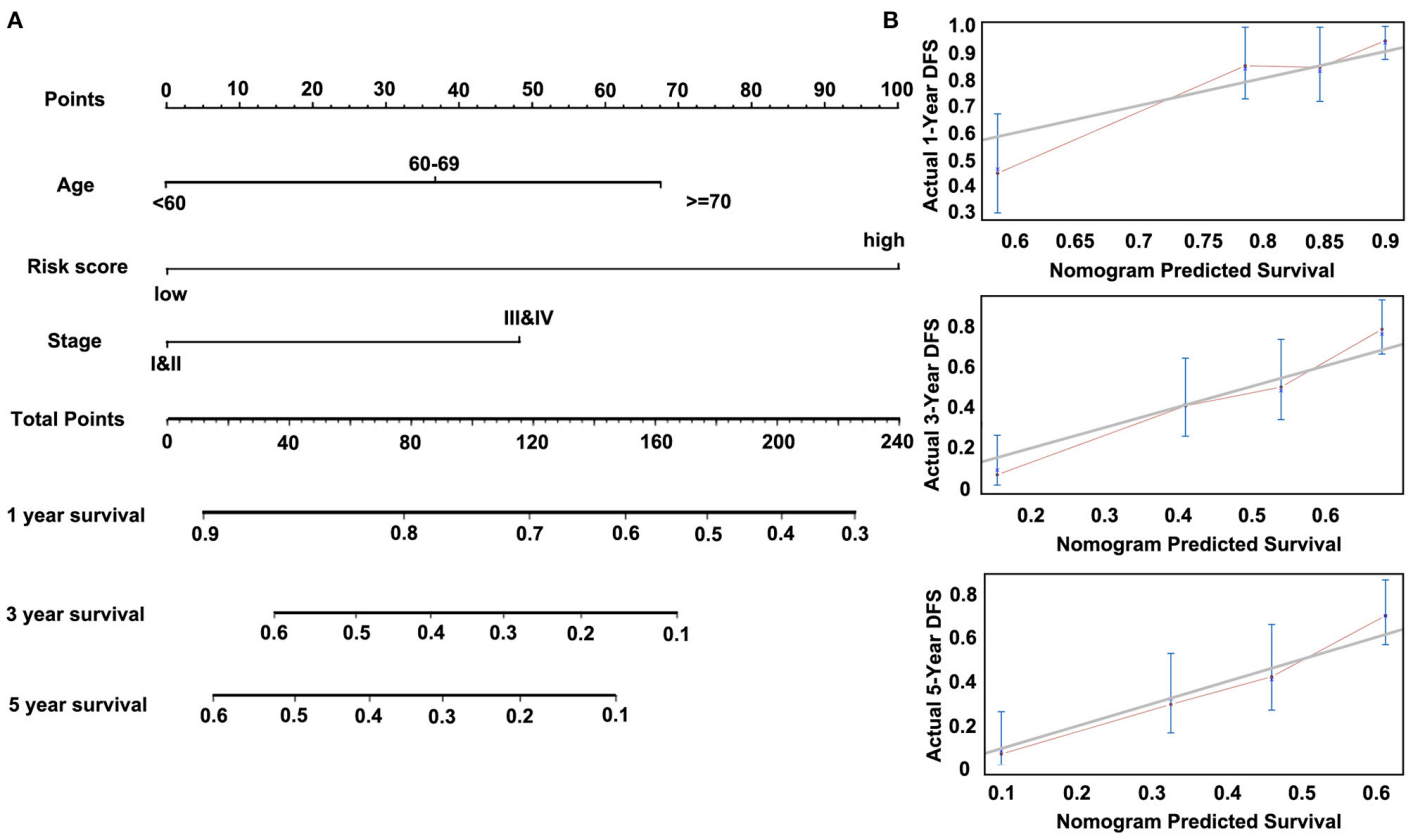
Nomogram of Cox regression model in the discovery cohort (GSE53624). **(A)** The nomogram for predicting overall survival (OS). **(B)** The calibration plots for predicting 1-, 3-, and 5-year OS.

Then, we constructed a nomogram model, as shown in Figure 7A. The univariate analysis was performed among nine variables to verify the prognostic variables with the data from the discovery cohort. Of the nine variables, a total of four variables were prognostic predictors for OS (including Age, Stage, Lymph node metastasis, and Risk score; *P* < 0.05). Three significant factors including Age, Stage (it is associated with Lymph node metastasis), and Risk score in the univariable analysis were enrolled into the multivariable analysis based on the Cox regression. A nomogram that incorporated the mentioned three prognostic factors was established. The prediction accuracy of the nomogram was assessed by C-index, and the results showed that the C-index was 0.713. To read the nomogram, draw a vertical line up to the top row of points to specify points for each variable. Then, the total points for a patient can be added up, and one can obtain the probability of 1-, 3-, and 5-year OS by drawing a vertical line from the total points row. Figure 7B showed the 1-, 3-, and 5-year nomogram model and the ideal model, and the results showed that the nomogram model was basically consistent with those of the ideal model. The nomogram was validated in the validation cohort, and 1- and 3-year calibration curves were presented in Supplementary Figure 5B. These results indicated that the accuracy of our model is relatively high.

### Analysis of Pathway Differences Enriched in the High-Risk and Low-Risk Groups

**Figure 8:**
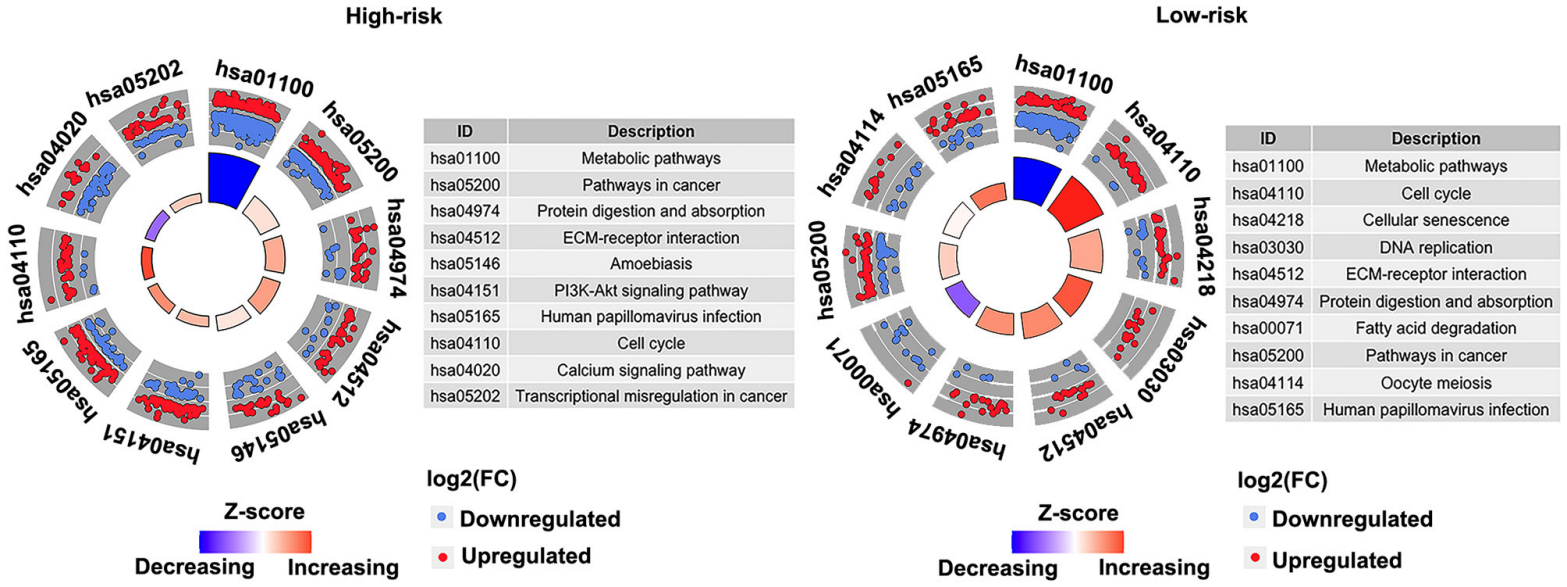
Kyoto Encyclopedia of Genes and Genomes (KEGG) pathways enriched in the high- and low-risk groups in the discovery cohort (GSE53624). The outer circle shows a scatter plot for each term of the log2(FC) of the assigned genes. Red circles display upregulation, and the blue ones display downregulation. Left, high-risk. Right, low-risk.

Pathway enrichment analysis of the DEGs in the high-risk group and the low-risk group showed that Metabolic pathways, Pathways in cancer, Protein digestion and absorption, Human papillomavirus infection, ECM–receptor interaction, and Cell cycle were enriched in both high-risk and low-risk groups. And results revealed that the high-risk may be related to the activity of PI3K–AKT signaling pathway and calcium signaling pathway, etc. (Figure 8). Besides, the activity of DNA replication and Fatty acid degradation may be related to low-risk (Figure 8). The suppression of the PI3K/AKT/mTOR signaling pathway can induce autophagy, which in turn saves tumor cells from the harm of epidermal growth factor receptor (EGFR)-tyrosine kinase inhibitors (TKIs) (20). Therefore, the PI3K–AKT pathway inhibitors may have potential targeting effect on patients in the high-risk group.

## Discussion

Cancers are highly heterogeneous diseases in that survival times vary substantially among patients with similar TNM stages. With the diagnosis and treatment at earlier stage, traditional clinicopathological indicators such as Tumor size, TNM stage, and Vascular invasion have proven inadequate for predicting individual prognosis (28). Since autophagy may play an important role in the development, progression, and therapeutic response of ESCC individually, the screening of prognostic molecular markers based on ARGs may reflect the biological characteristics of ESCC, which is of great significance for individualized prevention and treatment. To capture the genes necessary for ESCC from the perspective of autophagy, we screened ARGs and identified key prognostic ARGs, all of which may provide additional potential therapeutic targets. We further used the complementary value of molecular and clinical features and showed that combined analysis can provide a more accurate estimation of OS in ESCC. This comprehensive study of two factors contributes to our new understanding of ESCC biology and depicts potential therapeutic interventions.

In recent research, polygenic prognosis prediction models have been highlighted in clinical practice. For example, Oncotype DX, which provides a breast cancer recurrence score based on 21 genes (29–31), and Coloprin, which provides a colon cancer recurrence score based on 18 genes (32–34). These studies have shown that polygenic prognosis prediction models based on gene expression profiles are efficacious and promising to diagnosis, appropriate treatment, and improved prognosis of patients with cancer. Furthermore, Tian et al. (35) identified a six-gene signature, Zhao et al. (36) identified a three-gene signature, and Wang et al. (28) identified a six-gene signature. These signatures are proofs that the model composed of a small number of genes still has a high prediction efficiency of prognosis. In addition, there are some studies that identified signatures based on differentially expressed ARGs, 22-gene signature in non-small-cell lung cancer (NSCLC; 8) and three-gene signature in bladder cancer [BC; (37)]. These showed us that the model screened based on specific functions also has good efficiency and has good clinical application prospect. Based on these conditions, we screened three ARGs and constructed a polygenic prognosis prediction model and verified its predictive ability.

The three genes in our signature include PARP1, ITGA6, FADD as risk factors. PARP1 is a 113-kDa nuclear polymerase that modifies substrates (38). At present, it has been shown that PARP1 plays a role in the repair of DNA damage (39–41). PARP1-mediated autophagy is a key pathway for TKI resistance in NSCLC cells that participates in the resistance to TKIs (42). PARP1 may be an independent prognostic marker in ESCC, and PARP1 inhibition can induce cell cycle arrest at the G2/M phase through the ATM–Chk2–CDC25C pathway (38). ITGA6 is a member of the integrins family. Many integrins contribute to tumor progression, and ITGA6 has been implicated in breast cancer progression (43–45). In ESCC, it has been reported that expression of ITGA6 is highly upregulated and plays an important role in the proliferation and invasion (46). FADD is an adaptor molecule that interacts with various cell surface receptors and mediates cell apoptotic signals (47). In recent studies, FADD has been used as a potential autophagy-related prognostic marker in lung squamous cell carcinoma and head and neck squamous cell carcinoma (48, 49). The copy number amplification and upregulation of FADD were also found in ESCC, and its expression was significantly correlated with the survival of ESCC (50). Based on the PARP1–ITGA6–FADD three-gene model, ESCC patients were divided into high-risk group and low-risk group. Compared with single-gene prognosis analysis, this grouping method has more significant difference in prognosis. In addition, through KEGG enrichment analysis, we found that PI3K–AKT pathway was significantly enriched in the high-risk group. It has been proven that PI3K/AKT/mTOR-mediated autophagy played pivotal roles in the occurrence, development, and drug resistance of tumors. Autophagy mediated by PI3K–AKT–mTOR pathway can improve the drug sensitivity of tumor cells and avoid drug resistance (51). Therefore, ESCC patients in this high-risk group may benefit more from the targeted drugs. Through targeting PI3K–AKT–mTOR-mediated autophagy, many drugs can more accurately and specifically regulate autophagy activity of tumor cells, so as to achieve better antitumor therapeutic efficacy.

Lastly, we developed a nomogram to predict individuals' clinical outcomes. A nomogram is a stable and reliable tool to quantitatively measure risk on an individual basis by combining delineated risk factors, which has been used for autophagy prognoses (8). A nomogram generates a statistical predictive model presented in a graph, conferring points to each factor such as Stage, Grade, Age, and Gender in the clinical setting. By integrating all the factors, the model provides a predictive assessment for individuals. Apart from traditional clinicopathological features, the risk score based on genes can also be included in the predictive nomogram to better predict clinical results (52–54). Mo et al. (53) built a nomogram to predict survival in colorectal cancer with the inclusion of a prognostic score calculated from autophagy genes. Liu et al. (8) built a nomogram in non-small-cell lung cancer including a 22-autophagy gene signature that can well predict 3- and 5-year survival possibilities. In many cases, the combination of autophagy genes and prognostic factors has better prognosis than using a single factor. Moreover, we also used a calibration curve, the nomogram adopting both the gene signature and conventional prognostic factors that can accurately predict 3- and 5-year survival probabilities.

Although we have identified potential candidate genes and constructed a prognostic model using bioinformatics technology with ESCC samples, our study has several limitations. First, due to the lack of large public ESCC transcriptomic data, the sample size included in this study was not enough, which may affect the efficacy of our prognostic model. Second, although we have verified our findings in different cohorts, it would be better to confirm these results *via* independent experiments, such as immunohistochemistry in another cohort. Therefore, further genetic and experimental studies with larger samples and experimental validation are needed.

In conclusion, we divided ESCC patients into three clusters based on ARGs, and these clusters were related to stage and prognosis. Furthermore, we identified a prognostic three-autophagy gene signature base on GEO and TCGA ESCC cohorts. This three-gene model was an independent predictor of prognosis. And we used gene signature and clinicopathological features to build a nomogram that can accurately predict a 1-, 3-, and 5-year survival probability for individual ESCC patients. This finding suggests that the three-ARG signature may help facilitate personalized treatment.

## Data Availability Statement

The datasets presented in this study can be found in online repositories. The names of the repository/repositories and accession number(s) can be found in the article/Supplementary Material.

## Ethics Statement

Written informed consent was not obtained from the individual(s) for the publication of any potentially identifiable images or data included in this article.

## Author Contributions

HC, YZ, LW, and YW collated and analyzed the data. ND, CC, and LZ completed the writing and repair of the manuscript. WZ and YC designed and guided the subject. All authors reviewed and approved the final manuscript.

## Conflict of Interest

The authors declare that the research was conducted in the absence of any commercial or financial relationships that could be construed as a potential conflict of interest.

## Abbreviations

EIF2AK2: Eukaryotic Translation Initiation Factor 2 Alpha Kinase 2
BIRC5: Baculoviral IAP Repeat Containing 5
HSP90AB1: Heat Shock Protein 90 Alpha Family Class B Member 1
BID: BH3 Interacting Domain Death Agonist
GAA: Alpha Glucosidase
TP63: Tumor Protein P63
ITGB4: Integrin Subunit Beta 4
ITGB3: Integrin Subunit Beta 3
ATIC: 5-Aminoimidazole-4-Carboxamide Ribonucleotide Formyltransferase/IMP Cyclohydrolase
PELP1: Proline, Glutamate And Leucine Rich Protein 1
DDIT3: DNA Damage Inducible Transcript 3
PIK3R4: Phosphoinositide-3-Kinase Regulatory Subunit 4
SPNS1: Sphingolipid Transporter 1
CAPNS1: Calpain Small Subunit 1
FOXO3: Forkhead Box O3
SESN2: Sestrin 2
PARK2: Parkin RBR E3 Ubiquitin Protein Ligase
GNAI3: G Protein Subunit Alpha I3
SH3GLB1: SH3 Domain Containing GRB2 Like, Endophilin B1
ATG9B: Autophagy Related 9B
ULK3: Unc-51 Like Kinase 3
PINK1: PTEN Induced Kinase 1
ERO1L: Endoplasmic Reticulum Oxidoreductase 1 Alpha
CHMP2B: Charged Multivesicular Body Protein 2B
TP53INP2: Tumor Protein P53 Inducible Nuclear Protein 2
RAB11A: Member RAS Oncogene Family
NRG2: Neuregulin 2
ERBB2: Erb-B2 Receptor Tyrosine Kinase 2
MAPK3: Mitogen-Activated Protein Kinase 3
RAB5A: Member RAS Oncogene Family
HSPB8: Heat Shock Protein Family B (Small) Member 8.
