# Supplementary material for: Autophagy-Related Three-Gene Prognostic Signature for Predicting Survival in Esophageal Squamous Cell Carcinoma
Source: Front Oncol. 2021 Jul 15;11:650891. doi: 10.3389/fonc.2021.650891 (PMC8321089; doi:10.3389/fonc.2021.650891)
Supplement: Supplementary file 1 [file Data_Sheet_1.pdf]

Supplementary Figures and Figure Legends

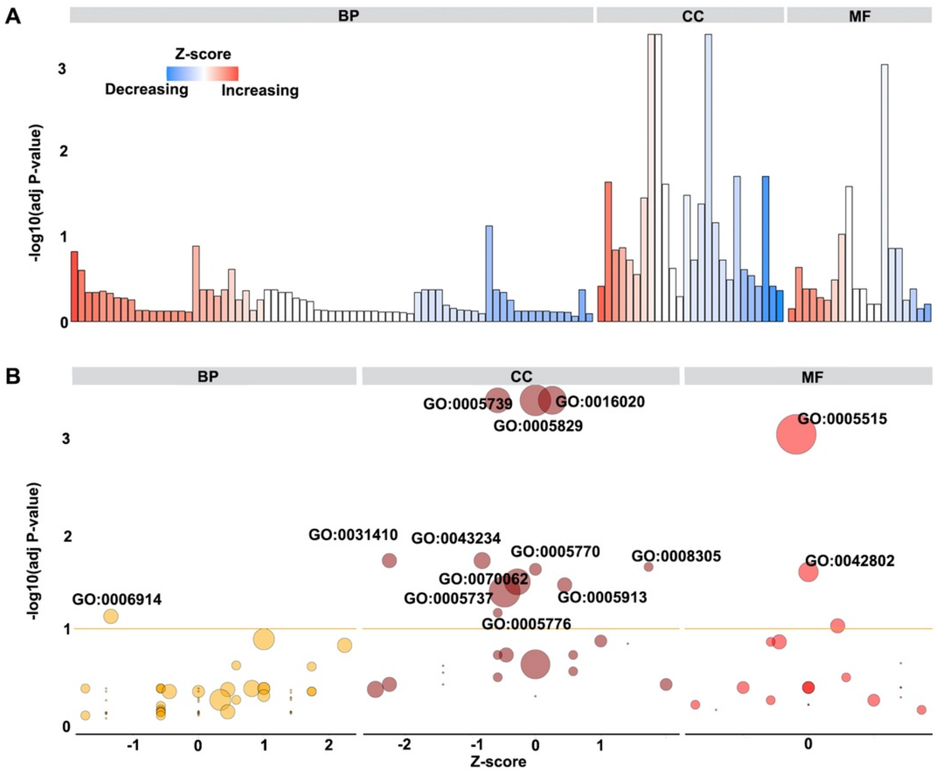

Supplementary Figure 1. The GO enrichment of differentially expressed autophagy-related genes (ARGs) in discovery cohort (GSE53624). (A) The bar chart of enriched gene ontology (GO) terms. The negative logarithm of the P-value to the y-axis and the GO terms are assigned to the x-axis. The bar color depth represents Z-score. **(B)** The bubble plot of enriched gene ontology (GO) terms. The Z-score is assigned to the x-axis, and the negative logarithm of the P-value to the y-axis, as in the barplot (the higher the more significant). The size of the displayed circles is proportional to the number of genes assigned to the term. BP, Biological process. CC, Cell component. MF, molecular function.

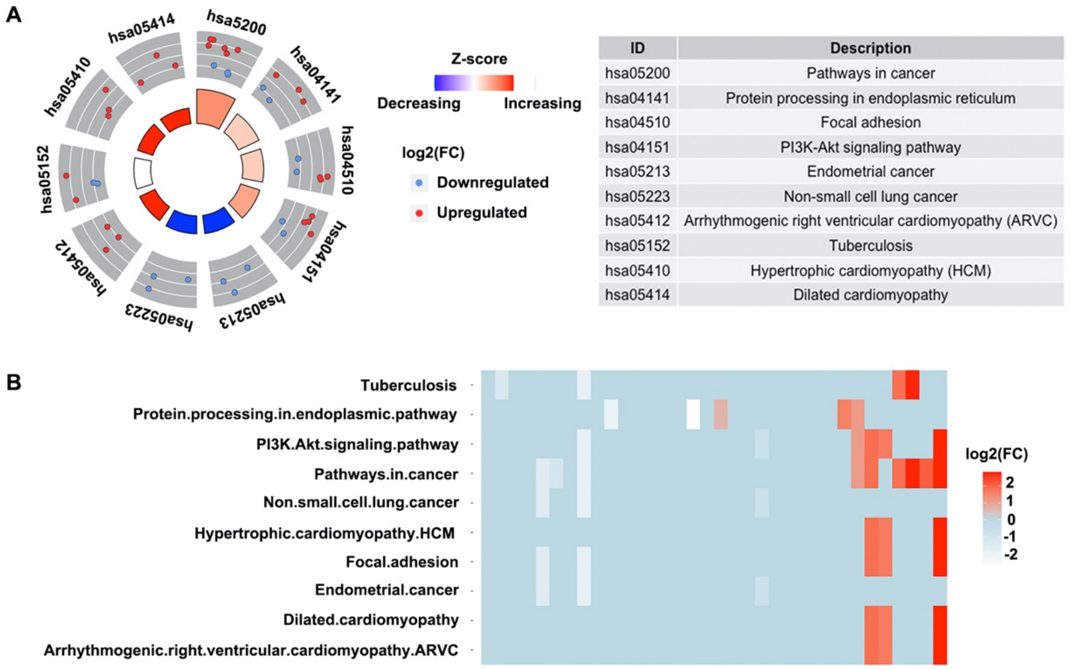

Supplementary Figure 2. The KEGG enrichment of differentially expressed ARGs in discovery cohort (GSE53624). Kyoto Encyclopedia of Genes and Genomes analysis of differentially expressed ARGs. **(A)** The outer circle shows a scatter plot for each term of the log2(FC) of the assigned genes. Red circles display up-regulation, and blue ones down-regulation. **(B)** The heatmap of the relationship between ARGs and pathways. The color of each block depends on the log2(FC) values.

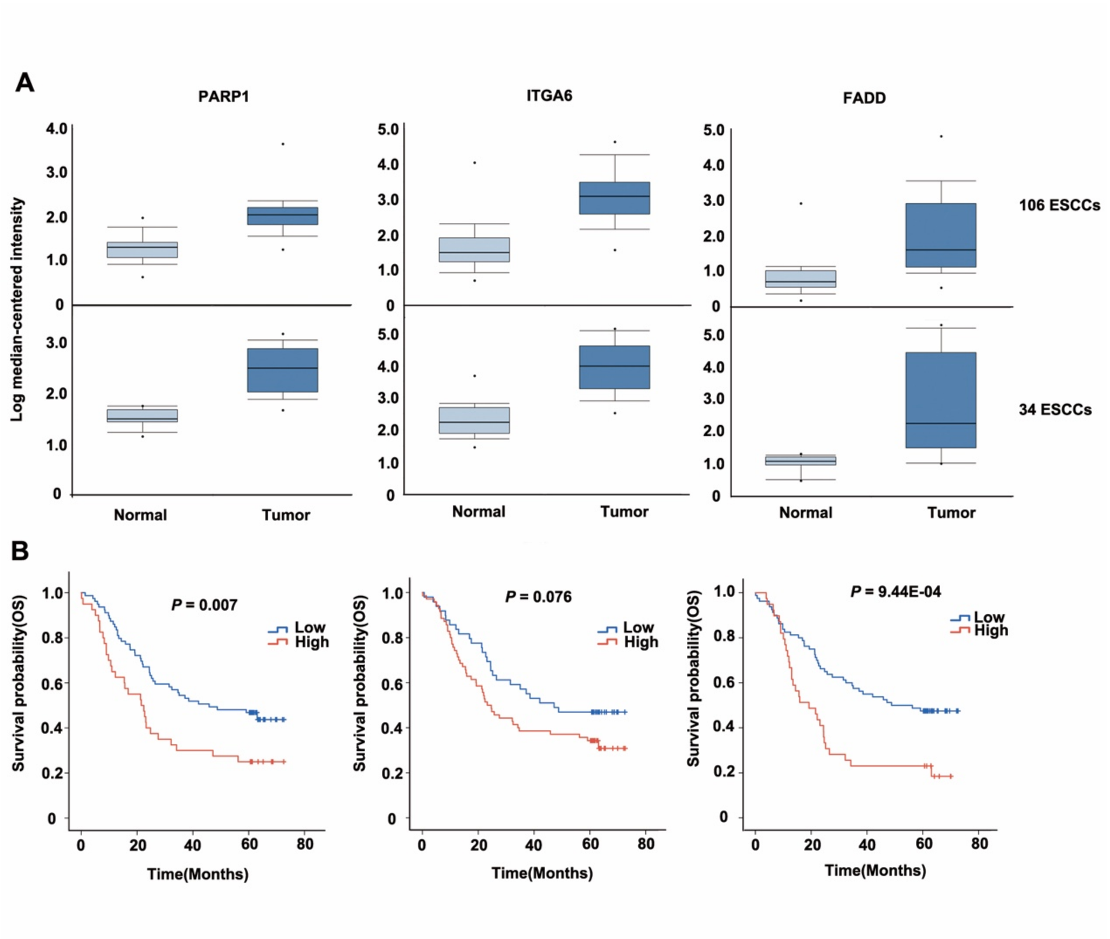

Supplementary Figure 3. 3-gene can be used as independent potential markers of ESCC. (A) Expression of 3 genes in two ESCC data from oncomine database. **(B)** Survival curve and Kaplan-Meier analysis of 3 genes in discovery cohort (GSE53624). OS, Overall Survival.

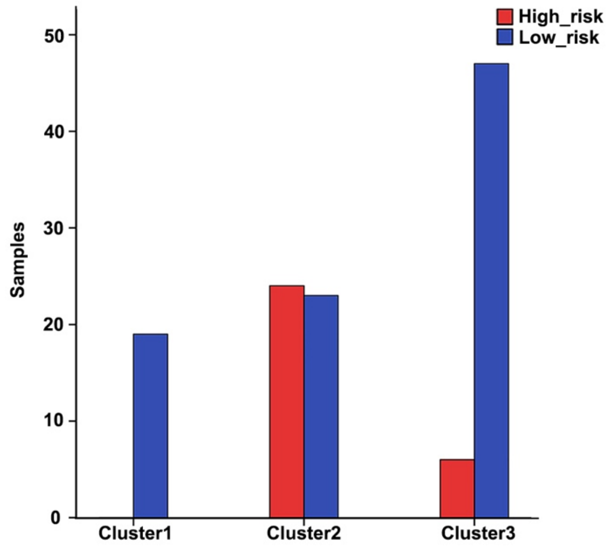

Supplementary Figure 4. Risk grouping histogram of the 3-gene signature model among three clusters in discovery cohort (GSE53624).

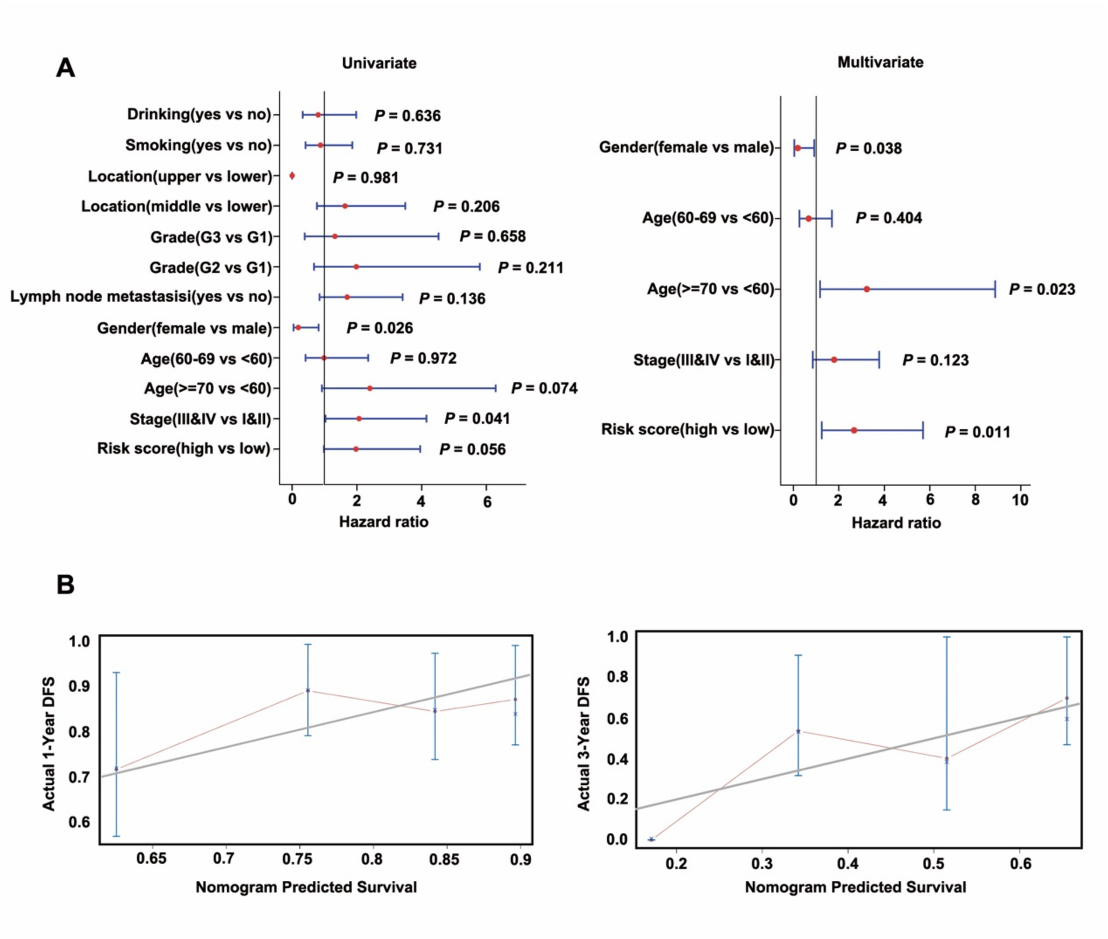

Supplementary Figure 5. Multi-analysis validation of 3-gene signature model in validation cohort (TCGA data). (A) Forest plot of Univariate and multivariate COX regression analyses. **(B)** The calibration plots of nomogram for predicting 1-, 3-years. DFS, Disease-free survival.
