# Supplementary material for: Autophagy-Related Three-Gene Prognostic Signature for Predicting Survival in Esophageal Squamous Cell Carcinoma
Source: Front Oncol. 2021 Jul 15;11:650891. doi: 10.3389/fonc.2021.650891 (PMC8321089; doi:10.3389/fonc.2021.650891)
Supplement: Supplementary file 2 [file Data_Sheet_2.docx]

| **Supplementary Table 1. Autophagy Related genes (ARGs) investigated in this study.** | | |
| --- | --- | --- |
| Gene symbol | Gene ID | Name |
| AMBRA1 | ENSG00000110497 | autophagy and beclin 1 regulator 1 |
| APOL1 | ENSG00000100342 | apolipoprotein L1 |
| ARNT | ENSG00000143437 | aryl hydrocarbon receptor nuclear translocator |
| ARSA | ENSG00000100299 | arylsulfatase A |
| ARSB | ENSG00000113273 | arylsulfatase B |
| ATF4 | ENSG00000128272 | activating transcription factor 4 |
| ATF6 | ENSG00000118217 | activating transcription factor 6 |
| ATG10 | ENSG00000152348 | autophagy related 10 |
| ATG101 | ENSG00000123395 | autophagy related 101 |
| ATG12 | ENSG00000145782 | autophagy related 12 |
| ATG13 | ENSG00000175224 | autophagy related 13 |
| ATG14 | ENSG00000126775 | autophagy related 14 |
| ATG16L1 | ENSG00000085978 | autophagy related 16 like 1 |
| ATG16L2 | ENSG00000168010 | autophagy related 16 like 2 |
| ATG2A | ENSG00000110046 | autophagy related 2A |
| ATG2B | ENSG00000066739 | autophagy related 2B |
| ATG3 | ENSG00000144848 | autophagy related 3 |
| ATG4A | ENSG00000101844 | autophagy related 4A cysteine peptidase |
| ATG4B | ENSG00000168397 | autophagy related 4B cysteine peptidase |
| ATG4C | ENSG00000125703 | autophagy related 4C cysteine peptidase |
| ATG4D | ENSG00000130734 | autophagy related 4D cysteine peptidase |
| ATG5 | ENSG00000057663 | autophagy related 5 |
| ATG7 | ENSG00000197548 | autophagy related 7 |
| ATG9A | ENSG00000198925 | autophagy related 9A |
| ATG9B | ENSG00000181652 | autophagy related 9B |
| ATIC | ENSG00000138363 | 5-aminoimidazole-4-carboxamide ribonucleotide formyltransferase/IMP cyclohydrolase |
| BAG1 | ENSG00000107262 | BCL2 associated athanogene 1 |
| BAG3 | ENSG00000151929 | BCL2 associated athanogene 3 |
| BAK1 | ENSG00000030110 | BCL2 antagonist/killer 1 |
| BAX | ENSG00000087088 | BCL2 associated X, apoptosis regulator |
| BCL2 | ENSG00000171791 | BCL2, apoptosis regulator |
| BCL2L1 | ENSG00000171552 | BCL2 like 1 |
| BECN1 | ENSG00000126581 | beclin 1 |
| BID | ENSG00000015475 | BH3 interacting domain death agonist |
| BIRC5 | ENSG00000089685 | baculoviral IAP repeat containing 5 |
| BIRC6 | ENSG00000115760 | baculoviral IAP repeat containing 6 |
| BNIP1 | ENSG00000113734 | BCL2 interacting protein 1 |
| BNIP3 | ENSG00000176171 | BCL2 interacting protein 3 |
| BNIP3L | ENSG00000104765 | BCL2 interacting protein 3 like |
| CALCOCO2 | ENSG00000136436 | calcium binding and coiled-coil domain 2 |
| CAMKK2 | ENSG00000110931 | calcium/calmodulin dependent protein kinase kinase 2 |
| CANX | ENSG00000127022 | calnexin |
| CAPN1 | ENSG00000014216 | calpain 1 |
| CAPN10 | ENSG00000142330 | calpain 10 |
| CAPN2 | ENSG00000162909 | calpain 2 |
| CAPNS1 | ENSG00000126247 | calpain small subunit 1 |
| CASP1 | ENSG00000137752 | caspase 1 |
| CASP3 | ENSG00000164305 | caspase 3 |
| CASP4 | ENSG00000196954 | caspase 4 |
| CASP8 | ENSG00000064012 | caspase 8 |
| CCL2 | ENSG00000108691 | C-C motif chemokine ligand 2 |
| CCR2 | ENSG00000121807 | C-C motif chemokine receptor 2 |
| CD46 | ENSG00000117335 | CD46 molecule |
| CDKN1A | ENSG00000124762 | cyclin dependent kinase inhibitor 1A |
| CDKN1B | ENSG00000111276 | cyclin dependent kinase inhibitor 1B |
| CDKN2A | ENSG00000147889 | cyclin dependent kinase inhibitor 2A |
| CFLAR | ENSG00000003402 | CASP8 and FADD like apoptosis regulator |
| CHMP2B | ENSG00000083937 | charged multivesicular body protein 2B |
| CHMP4B | ENSG00000101421 | charged multivesicular body protein 4B |
| CLN3 | ENSG00000188603 | CLN3, battenin |
| CTSB | ENSG00000164733 | cathepsin B |
| CTSD | ENSG00000117984 | cathepsin D |
| CTSL | ENSG00000135047 | cathepsin L |
| CX3CL1 | ENSG00000006210 | C-X3-C motif chemokine ligand 1 |
| CXCR4 | ENSG00000121966 | C-X-C motif chemokine receptor 4 |
| DAPK1 | ENSG00000196730 | death associated protein kinase 1 |
| DAPK2 | ENSG00000035664 | death associated protein kinase 2 |
| DDIT3 | ENSG00000175197 | DNA damage inducible transcript 3 |
| DIRAS3 | ENSG00000162595 | DIRAS family GTPase 3 |
| DLC1 | ENSG00000164741 | DLC1 Rho GTPase activating protein |
| DNAJB1 | ENSG00000132002 | DnaJ heat shock protein family (Hsp40) member B1 |
| DNAJB9 | ENSG00000128590 | DnaJ heat shock protein family (Hsp40) member B9 |
| DRAM1 | ENSG00000136048 | DNA damage regulated autophagy modulator 1 |
| EDEM1 | ENSG00000134109 | ER degradation enhancing alpha-mannosidase like protein 1 |
| EEF2 | ENSG00000167658 | eukaryotic translation elongation factor 2 |
| EEF2K | ENSG00000103319 | eukaryotic elongation factor 2 kinase |
| EGFR | ENSG00000146648 | epidermal growth factor receptor |
| EIF2AK2 | ENSG00000055332 | eukaryotic translation initiation factor 2 alpha kinase 2 |
| EIF2AK3 | ENSG00000172071 | eukaryotic translation initiation factor 2 alpha kinase 3 |
| EIF2S1 | ENSG00000134001 | eukaryotic translation initiation factor 2 subunit alpha |
| EIF4EBP1 | ENSG00000187840 | eukaryotic translation initiation factor 4E binding protein 1 |
| EIF4G1 | ENSG00000114867 | eukaryotic translation initiation factor 4 gamma 1 |
| ERBB2 | ENSG00000141736 | erb-b2 receptor tyrosine kinase 2 |
| ERN1 | ENSG00000178607 | endoplasmic reticulum to nucleus signaling 1 |
| ERO1A | ENSG00000197930 | endoplasmic reticulum oxidoreductase 1 alpha |
| FADD | ENSG00000168040 | Fas associated via death domain |
| FAM215A | ENSG00000267496 | family with sequence similarity 215 member A (non-protein coding) |
| FAS | ENSG00000026103 | Fas cell surface death receptor |
| FKBP1A | ENSG00000088832 | FK506 binding protein 1A |
| FKBP1B | ENSG00000119782 | FK506 binding protein 1B |
| FOS | ENSG00000170345 | Fos proto-oncogene, AP-1 transcription factor subunit |
| FOXO1 | ENSG00000150907 | forkhead box O1 |
| FOXO3 | ENSG00000118689 | forkhead box O3 |
| GAA | ENSG00000171298 | glucosidase alpha, acid |
| GABARAP | ENSG00000170296 | GABA type A receptor-associated protein |
| GABARAPL1 | ENSG00000139112 | GABA type A receptor associated protein like 1 |
| GABARAPL2 | ENSG00000034713 | GABA type A receptor associated protein like 2 |
| GAPDH | ENSG00000111640 | glyceraldehyde-3-phosphate dehydrogenase |
| GNAI3 | ENSG00000065135 | G protein subunit alpha i3 |
| GOPC | ENSG00000047932 | golgi associated PDZ and coiled-coil motif containing |
| GRID1 | ENSG00000182771 | glutamate ionotropic receptor delta type subunit 1 |
| GRID2 | ENSG00000152208 | glutamate ionotropic receptor delta type subunit 2 |
| HDAC1 | ENSG00000116478 | histone deacetylase 1 |
| HDAC6 | ENSG00000094631 | histone deacetylase 6 |
| HGS | ENSG00000185359 | hepatocyte growth factor-regulated tyrosine kinase substrate |
| HIF1A | ENSG00000100644 | hypoxia inducible factor 1 alpha subunit |
| HSP90AB1 | ENSG00000096384 | heat shock protein 90 alpha family class B member 1 |
| HSPA5 | ENSG00000044574 | heat shock protein family A (Hsp70) member 5 |
| HSPA8 | ENSG00000109971 | heat shock protein family A (Hsp70) member 8 |
| HSPB8 | ENSG00000152137 | heat shock protein family B (small) member 8 |
| IFNG | ENSG00000111537 | interferon gamma |
| IKBKB | ENSG00000104365 | inhibitor of nuclear factor kappa B kinase subunit beta |
| IKBKE | ENSG00000263528 | inhibitor of nuclear factor kappa B kinase subunit epsilon |
| IL24 | ENSG00000162892 | interleukin 24 |
| IRGM | ENSG00000237693 | immunity related GTPase M |
| ITGA3 | ENSG00000005884 | integrin subunit alpha 3 |
| ITGA6 | ENSG00000091409 | integrin subunit alpha 6 |
| ITGB1 | ENSG00000150093 | integrin subunit beta 1 |
| ITGB4 | ENSG00000132470 | integrin subunit beta 4 |
| ITPR1 | ENSG00000150995 | inositol 1,4,5-trisphosphate receptor type 1 |
| KIF5B | ENSG00000170759 | kinesin family member 5B |
| KLHL24 | ENSG00000114796 | kelch like family member 24 |
| LAMP1 | ENSG00000185896 | lysosomal associated membrane protein 1 |
| LAMP2 | ENSG00000005893 | lysosomal associated membrane protein 2 |
| MAP1LC3A | ENSG00000101460 | microtubule associated protein 1 light chain 3 alpha |
| MAP1LC3B | ENSG00000140941 | microtubule associated protein 1 light chain 3 beta |
| MAP1LC3C | ENSG00000197769 | microtubule associated protein 1 light chain 3 gamma |
| MAP2K7 | ENSG00000076984 | mitogen-activated protein kinase kinase 7 |
| MAPK1 | ENSG00000100030 | mitogen-activated protein kinase 1 |
| MAPK3 | ENSG00000102882 | mitogen-activated protein kinase 3 |
| MAPK8 | ENSG00000107643 | mitogen-activated protein kinase 8 |
| MAPK8IP1 | ENSG00000121653 | mitogen-activated protein kinase 8 interacting protein 1 |
| MAPK9 | ENSG00000050748 | mitogen-activated protein kinase 9 |
| MBTPS2 | ENSG00000012174 | membrane bound transcription factor peptidase, site 2 |
| MLST8 | ENSG00000167965 | MTOR associated protein, LST8 homolog |
| MTMR14 | ENSG00000163719 | myotubularin related protein 14 |
| MTOR | ENSG00000198793 | mechanistic target of rapamycin kinase |
| MYC | ENSG00000136997 | MYC proto-oncogene, bHLH transcription factor |
| NAF1 | ENSG00000145414 | nuclear assembly factor 1 ribonucleoprotein |
| NAMPT | ENSG00000105835 | nicotinamide phosphoribosyltransferase |
| NBR1 | ENSG00000188554 | NBR1, autophagy cargo receptor |
| NCKAP1 | ENSG00000061676 | NCK associated protein 1 |
| NFE2L2 | ENSG00000116044 | nuclear factor, erythroid 2 like 2 |
| NFKB1 | ENSG00000109320 | nuclear factor kappa B subunit 1 |
| NKX2-3 | ENSG00000119919 | NK2 homeobox 3 |
| NLRC4 | ENSG00000091106 | NLR family CARD domain containing 4 |
| NPC1 | ENSG00000141458 | NPC intracellular cholesterol transporter 1 |
| NRG1 | ENSG00000157168 | neuregulin 1 |
| NRG2 | ENSG00000158458 | neuregulin 2 |
| NRG3 | ENSG00000185737 | neuregulin 3 |
| P4HB | ENSG00000185624 | prolyl 4-hydroxylase subunit beta |
| PARP1 | ENSG00000143799 | poly(ADP-ribose) polymerase 1 |
| PEA15 | ENSG00000162734 | proliferation and apoptosis adaptor protein 15 |
| PELP1 | ENSG00000141456 | proline, glutamate and leucine rich protein 1 |
| PEX14 | ENSG00000142655 | peroxisomal biogenesis factor 14 |
| PEX3 | ENSG00000034693 | peroxisomal biogenesis factor 3 |
| PIK3C3 | ENSG00000078142 | phosphatidylinositol 3-kinase catalytic subunit type 3 |
| PIK3R4 | ENSG00000196455 | phosphoinositide-3-kinase regulatory subunit 4 |
| PINK1 | ENSG00000158828 | PTEN induced putative kinase 1 |
| PPP1R15A | ENSG00000087074 | protein phosphatase 1 regulatory subunit 15A |
| PRKAB1 | ENSG00000111725 | protein kinase AMP-activated non-catalytic subunit beta 1 |
| PRKAR1A | ENSG00000108946 | protein kinase cAMP-dependent type I regulatory subunit alpha |
| PRKCD | ENSG00000163932 | protein kinase C delta |
| PRKCQ | ENSG00000065675 | protein kinase C theta |
| PRKN | ENSG00000185345 | parkin RBR E3 ubiquitin protein ligase |
| PTEN | ENSG00000171862 | phosphatase and tensin homolog |
| PTK6 | ENSG00000101213 | protein tyrosine kinase 6 |
| RAB11A | ENSG00000103769 | RAB11A, member RAS oncogene family |
| RAB1A | ENSG00000138069 | RAB1A, member RAS oncogene family |
| RAB24 | ENSG00000169228 | RAB24, member RAS oncogene family |
| RAB33B | ENSG00000172007 | RAB33B, member RAS oncogene family |
| RAB5A | ENSG00000144566 | RAB5A, member RAS oncogene family |
| RAB7A | ENSG00000075785 | RAB7A, member RAS oncogene family |
| RAC1 | ENSG00000136238 | Rac family small GTPase 1 |
| RACK1 | ENSG00000204628 | receptor for activated C kinase 1 |
| RAF1 | ENSG00000132155 | Raf-1 proto-oncogene, serine/threonine kinase |
| RB1 | ENSG00000139687 | RB transcriptional corepressor 1 |
| RB1CC1 | ENSG00000023287 | RB1 inducible coiled-coil 1 |
| RELA | ENSG00000173039 | RELA proto-oncogene, NF-kB subunit |
| RGS19 | ENSG00000171700 | regulator of G protein signaling 19 |
| RHEB | ENSG00000106615 | Ras homolog, mTORC1 binding |
| RPS6KB1 | ENSG00000108443 | ribosomal protein S6 kinase B1 |
| RPTOR | ENSG00000141564 | regulatory associated protein of MTOR complex 1 |
| RUBCN | ENSG00000145016 | RUN and cysteine rich domain containing beclin 1 interacting protein |
| SAR1A | ENSG00000079332 | secretion associated Ras related GTPase 1A |
| SERPINA1 | ENSG00000197249 | serpin family A member 1 |
| SESN2 | ENSG00000130766 | sestrin 2 |
| SH3GLB1 | ENSG00000097033 | SH3 domain containing GRB2 like, endophilin B1 |
| SIRT1 | ENSG00000096717 | sirtuin 1 |
| SIRT2 | ENSG00000068903 | sirtuin 2 |
| SPHK1 | ENSG00000176170 | sphingosine kinase 1 |
| SPNS1 | ENSG00000169682 | sphingolipid transporter 1 (putative) |
| SQSTM1 | ENSG00000161011 | sequestosome 1 |
| ST13 | ENSG00000100380 | ST13, Hsp70 interacting protein |
| STK11 | ENSG00000118046 | serine/threonine kinase 11 |
| SUPT20H | ENSG00000102710 | SPT20 homolog, SAGA complex component |
| TBK1 | ENSG00000183735 | TANK binding kinase 1 |
| TM9SF1 | ENSG00000100926 | transmembrane 9 superfamily member 1 |
| TMEM74 | ENSG00000164841 | transmembrane protein 74 |
| TNFSF10 | ENSG00000121858 | TNF superfamily member 10 |
| TP53 | ENSG00000141510 | tumor protein p53 |
| TP53INP2 | ENSG00000078804 | tumor protein p53 inducible nuclear protein 2 |
| TP63 | ENSG00000073282 | tumor protein p63 |
| TP73 | ENSG00000078900 | tumor protein p73 |
| TSC1 | ENSG00000165699 | TSC complex subunit 1 |
| TSC2 | ENSG00000103197 | TSC complex subunit 2 |
| TUSC1 | ENSG00000198680 | tumor suppressor candidate 1 |
| ULK1 | ENSG00000177169 | unc-51 like autophagy activating kinase 1 |
| ULK2 | ENSG00000083290 | unc-51 like autophagy activating kinase 2 |
| ULK3 | ENSG00000140474 | unc-51 like kinase 3 |
| USP10 | ENSG00000103194 | ubiquitin specific peptidase 10 |
| UVRAG | ENSG00000198382 | UV radiation resistance associated |
| VAMP3 | ENSG00000049245 | vesicle associated membrane protein 3 |
| VAMP7 | ENSG00000124333 | vesicle associated membrane protein 7 |
| VEGFA | ENSG00000112715 | vascular endothelial growth factor A |
| VMP1 | ENSG00000062716 | vacuole membrane protein 1 |
| WDFY3 | ENSG00000163625 | WD repeat and FYVE domain containing 3 |
| WDR45 | ENSG00000196998 | WD repeat domain 45 |
| WDR45B | ENSG00000141580 | WD repeat domain 45B |
| WIPI1 | ENSG00000070540 | WD repeat domain, phosphoinositide interacting 1 |
| WIPI2 | ENSG00000157954 | WD repeat domain, phosphoinositide interacting 2 |
| ZFYVE1 | ENSG00000165861 | zinc finger FYVE-type containing 1 |

| **Supplementary Table 2. The differentially expressed genes were screened after the first step** | | | | |
| --- | --- | --- | --- | --- |
| GeneSymbol | ID | 119 pair ESCCs | | |
|  |  | t test q value | up/down | AUC |
| EIF2AK2 | ENSG00000055332 | 8.87074E-66 | up | 0.982557729 |
| BIRC5 | ENSG00000089685 | 1.34395E-49 | up | 0.964762376 |
| HSP90AB1 | ENSG00000096384 | 1.21689E-47 | up | 0.959183673 |
| BID | ENSG00000015475 | 3.40973E-43 | up | 0.949085517 |
| USP10 | ENSG00000103194 | 2.87032E-42 | up | 0.930372149 |
| ITGA6 | ENSG00000091409 | 1.11767E-36 | up | 0.924228515 |
| GAA | ENSG00000171298 | 5.41013E-35 | up | 0.917202175 |
| TP63 | ENSG00000073282 | 1.00454E-24 | up | 0.912470871 |
| ITGB4 | ENSG00000132470 | 1.50219E-32 | up | 0.910811383 |
| PARP1 | ENSG00000143799 | 2.96282E-31 | up | 0.898735965 |
| ITGA3 | ENSG00000005884 | 1.7794E-29 | up | 0.897606101 |
| ATIC | ENSG00000138363 | 2.73136E-30 | up | 0.894004661 |
| FADD | ENSG00000168040 | 4.80781E-30 | up | 0.887755102 |
| PELP1 | ENSG00000141456 | 1.56659E-27 | up | 0.880799379 |
| DDIT3 | ENSG00000175197 | 3.09179E-26 | up | 0.878539651 |
| PIK3R4 | ENSG00000196455 | 2.32944E-25 | up | 0.876633006 |
| SPNS1 | ENSG00000169682 | 1.98545E-22 | up | 0.850893298 |
| CAPNS1 | ENSG00000126247 | 7.41769E-25 | down | 0.86250971 |
| PRKCD | ENSG00000163932 | 2.33849E-25 | down | 0.864840054 |
| PEA15 | ENSG00000162734 | 2.17954E-26 | down | 0.871619236 |
| GABARAP | ENSG00000170296 | 2.57952E-24 | down | 0.878504343 |
| RAB24 | ENSG00000169228 | 3.92838E-28 | down | 0.883023798 |
| FOXO3 | ENSG00000118689 | 1.62936E-28 | down | 0.883906504 |
| SESN2 | ENSG00000130766 | 1.10476E-30 | down | 0.894640209 |
| NBR1 | ENSG00000188554 | 1.53846E-29 | down | 0.901984323 |
| ERN1 | ENSG00000178607 | 1.33196E-32 | down | 0.90826919 |
| PARK2 | ENSG00000185345 | 2.77552E-35 | down | 0.916390085 |
| GNAI3 | ENSG00000065135 | 1.4363E-34 | down | 0.920132759 |
| SH3GLB1 | ENSG00000097033 | 1.34815E-34 | down | 0.926488242 |
| ATG9B | ENSG00000181652 | 5.20135E-45 | down | 0.934397288 |
| ULK3 | ENSG00000140474 | 3.12822E-42 | down | 0.938846127 |
| PINK1 | ENSG00000158828 | 4.72852E-38 | down | 0.94318904 |
| ERO1L | ENSG00000197930 | 1.71448E-46 | down | 0.945978391 |
| CHMP2B | ENSG00000083937 | 1.21177E-37 | down | 0.946896406 |
| RAF1 | ENSG00000132155 | 3.09853E-44 | down | 0.950639079 |
| TP53INP2 | ENSG00000078804 | 7.06585E-48 | down | 0.950709696 |
| RAB11A | ENSG00000103769 | 2.71602E-44 | down | 0.954664219 |
| NRG2 | ENSG00000158458 | 2.46567E-50 | down | 0.955794082 |
| ERBB2 | ENSG00000141736 | 5.1762E-46 | down | 0.961372784 |
| MAPK3 | ENSG00000102882 | 2.58227E-56 | down | 0.966245322 |
| RAB5A | ENSG00000144566 | 1.9132E-54 | down | 0.970411694 |
| HSPB8 | ENSG00000152137 | 9.07055E-53 | down | 0.978461973 |

| **Supplementary Table 3. The final differentially expressed gene was verified** | | | | | | | | | | |
| --- | --- | --- | --- | --- | --- | --- | --- | --- | --- | --- |
| GeneSymbol | ID | Differential expression | | | | | | | Oncomine Validation | |
|  |  | 119 pair ESCCs | | | | | 155 pairs ESCCs | | 53 pairs ESCCs | |
|  |  | t test q value | up/down | padj | log2FC | AUC | log2FC | padj | P-value | log2FC |
| ITGA6 | ENSG00000091409 | 1.11767E-36 | up | 9.97E-31 | 2.86554747 | 0.92422851 | 1.8872286 | 2.17804E-85 | 6.03E-19 | 2.738 |
| FADD | ENSG00000168040 | 4.80781E-30 | up | 3.89E-18 | 2.82139897 | 0.8877551 | 2.02100221 | 3.94149E-65 | 2.80E-10 | 2.251 |
| BIRC5 | ENSG00000089685 | 1.34395E-49 | up | 6.07E-19 | 2.17590565 | 0.96476238 | 1.77997906 | 6.3402E-113 | 2.48E-22 | 2.631 |
| ITGA3 | ENSG00000005884 | 1.7794E-29 | up | 1.05E-13 | 1.89632038 | 0.8976061 | 1.44507789 | 8.89985E-51 | 2.72E-08 | 2.134 |
| BID | ENSG00000015475 | 3.40973E-43 | up | 3.89E-16 | 1.89299042 | 0.94908552 | 1.36591478 | 2.86625E-87 | 9.29E-19 | 1.467 |
| ITGB4 | ENSG00000132470 | 1.50219E-32 | up | 1.66E-16 | 1.7210344 | 0.91081138 | 1.42520509 | 4.07848E-69 | 1.20E-17 | 2.312 |
| TP63 | ENSG00000073282 | 1.00454E-24 | up | 3.99E-17 | 1.64036595 | 0.91247087 | 1.24239432 | 1.53382E-38 | 4.59E-16 | 3.032 |
| EIF2AK2 | ENSG00000055332 | 8.87074E-66 | up | 1.14E-23 | 1.62801005 | 0.98255773 | 1.08192698 | 7.10535E-69 | 8.65E-08 | 2.023 |
| HSP90AB1 | ENSG00000096384 | 1.21689E-47 | up | 3.28E-17 | 1.17322312 | 0.95918367 | 0.71113896 | 9.02914E-41 | 2.05E-23 | 1.662 |
| ATIC | ENSG00000138363 | 2.73136E-30 | up | 5.79E-15 | 1.05607908 | 0.89400466 | 0.6821415 | 3.73684E-17 | 5.45E-16 | 1.62 |
| SPNS1 | ENSG00000169682 | 1.98545E-22 | up | 1.02E-10 | 1.05107195 | 0.8508933 | 0.8908492 | 2.09063E-41 | 1.56E-07 | 1.238 |
| PARP1 | ENSG00000143799 | 2.96282E-31 | up | 8.71E-11 | 0.81381127 | 0.89873596 | 0.89417404 | 5.01E-82 | 6.35E-18 | 1.663 |
| GAA | ENSG00000171298 | 5.41013E-35 | up | 4.64E-08 | 0.80330755 | 0.91720217 | 1.0662006 | 7.49189E-48 | 7.16E-12 | 1.548 |
| PELP1 | ENSG00000141456 | 1.56659E-27 | up | 8.60E-11 | 0.75434562 | 0.88079938 | 0.60421201 | 5.47287E-21 | 5.49E-05 | 1.101 |
| DDIT3 | ENSG00000175197 | 3.09179E-26 | up | 2.51E-10 | 0.71594277 | 0.87853965 | 0.92329793 | 9.82791E-29 | 1.29E-01 | 1.041 |
| PIK3R4 | ENSG00000196455 | 2.32944E-25 | up | 5.56E-07 | 0.64975017 | 0.87663301 | 0.60194207 | 1.29776E-40 | 2.25E-14 | 1.594 |
| FOXO3 | ENSG00000118689 | 1.62936E-28 | down | 1.01E-07 | -0.6521636 | 0.8839065 | -0.5734861 | 1.25155E-27 | 1.21E-07 | -1.242 |
| CAPNS1 | ENSG00000126247 | 7.41769E-25 | down | 4.66E-09 | -0.8448707 | 0.86250971 | -0.8607683 | 1.39424E-38 | 2.59E-11 | -1.433 |
| GNAI3 | ENSG00000065135 | 1.4363E-34 | down | 1.29E-09 | -1.001134 | 0.92013276 | -0.6112458 | 1.18027E-39 | 1.63E-07 | -1.351 |
| SH3GLB1 | ENSG00000097033 | 1.34815E-34 | down | 2.22E-13 | -1.1131527 | 0.92648824 | -0.69453 | 1.99338E-57 | 8.06E-22 | -3.276 |
| PINK1 | ENSG00000158828 | 4.72852E-38 | down | 3.52E-11 | -1.1876498 | 0.94318904 | -0.9511151 | 2.33447E-57 | 4.33E-21 | -1.645 |
| CHMP2B | ENSG00000083937 | 1.21177E-37 | down | 4.19E-13 | -1.1995906 | 0.94689641 | -0.8503965 | 2.11489E-72 | 5.25E-16 | -1.415 |
| RAB5A | ENSG00000144566 | 1.9132E-54 | down | 2.03E-19 | -1.2229117 | 0.97041169 | -0.7812342 | 1.46943E-71 | 4.11E-14 | -1.715 |
| ERBB2 | ENSG00000141736 | 5.1762E-46 | down | 1.27E-18 | -1.5303729 | 0.96137278 | -0.8174579 | 2.36475E-18 | 2.82E-07 | -1.519 |
| SESN2 | ENSG00000130766 | 1.10476E-30 | down | 1.27E-11 | -1.6204683 | 0.89464021 | -1.325478 | 6.92867E-50 | 1.21E-15 | -1.754 |
| MAPK3 | ENSG00000102882 | 2.58227E-56 | down | 8.93E-22 | -1.8290431 | 0.96624532 | -1.4630692 | 1.38336E-95 | 8.29E-17 | -1.461 |
| ULK3 | ENSG00000140474 | 3.12822E-42 | down | 2.03E-18 | -1.8882506 | 0.93884613 | -1.2248504 | 2.46285E-78 | 6.83E-13 | -1.676 |
| RAB11A | ENSG00000103769 | 2.71602E-44 | down | 1.80E-19 | -1.9241102 | 0.95466422 | -1.2907548 | 7.0933E-141 | 1.52E-12 | -2.149 |
| PARK2 | ENSG00000185345 | 2.77552E-35 | down | 1.29E-07 | -2.024158 | 0.91639009 | -1.215319 | 4.26353E-36 | 5.72E-07 | -1.081 |
| TP53INP2 | ENSG00000078804 | 7.06585E-48 | down | 4.32E-27 | -2.4558901 | 0.9507097 | -1.5113989 | 2.58163E-37 | 2.71E-18 | -2.685 |
| ERO1L | ENSG00000197930 | 1.71448E-46 | down | 2.39E-22 | -2.7636093 | 0.94597839 | -2.0683322 | 1.60498E-79 | 1.55E-11 | -2.383 |
| NRG2 | ENSG00000158458 | 2.46567E-50 | down | 1.30E-13 | -3.0365722 | 0.95579408 | -2.5302672 | 1.93057E-68 | 9.38E-07 | -1.064 |
| HSPB8 | ENSG00000152137 | 9.07055E-53 | down | 3.55E-23 | -3.1110701 | 0.97846197 | -1.6807311 | 1.52076E-43 | 2.71E-17 | -3.109 |
| ATG9B | ENSG00000181652 | 5.20135E-45 | down | 2.37E-18 | -3.3772494 | 0.93439729 | -2.8069411 | 5.29466E-62 | 2.52E-16 | -2.77 |

| **Supplementary Table 4. The relationships between the subtypes and clinicopathological parameters in discovery cohort (GSE53624)** | | | | | | | | | |
| --- | --- | --- | --- | --- | --- | --- | --- | --- | --- |
| Clinical, epidemiological or pathological feature | | Total (N) | Cluster1 | Proportion | Cluster2 | Proportion | Cluster3 | Proportion | P-value |
|  |  |  |  |  |  |  |  |  |  |
|  |  |  |  |  |  |  |  |  |  |
| All cases |  | 119 | 19 |  | 47 |  | 53 |  |  |
| Age | <60 | 61 | 12 | 19.67% | 23 | 37.70% | 26 | 42.62% | 0.838 |
|  | 60-69 | 45 | 5 | 11.11% | 19 | 42.22% | 21 | 46.67% |  |
|  | ≥70 | 13 | 2 | 15.38% | 5 | 38.46% | 6 | 46.15% |  |
| Gender | Male | 98 | 16 | 16.33% | 40 | 40.82% | 42 | 42.86% | 0.725 |
|  | Female | 21 | 3 | 14.29% | 7 | 33.33% | 11 | 52.38% |  |
| Smoking | No | 39 | 7 | 17.95% | 13 | 33.33% | 19 | 48.72% | 0.629 |
|  | Yes | 80 | 12 | 15.00% | 34 | 42.50% | 34 | 42.50% |  |
| Drinking | No | 45 | 5 | 11.11% | 12 | 26.67% | 28 | 62.22% | 0.010 |
|  | Yes | 74 | 14 | 18.92% | 35 | 47.30% | 25 | 33.78% |  |
| Location | Upper thoracic | 14 | 2 | 14.29% | 7 | 50.00% | 5 | 35.71% | 0.092 |
|  | Middle thoracic | 69 | 12 | 17.39% | 20 | 28.99% | 37 | 53.62% |  |
|  | Lower thoracic | 36 | 5 | 13.89% | 20 | 55.56% | 11 | 30.56% |  |
| Grade | Grade1 | 23 | 6 | 26.09% | 9 | 39.13% | 8 | 34.78% | 0.009 |
|  | Grade2 | 64 | 12 | 18.75% | 29 | 45.31% | 23 | 35.94% |  |
|  | Grade3 | 32 | 1 | 3.13% | 9 | 28.13% | 22 | 68.75% |  |
| Stage | I&II | 53 | 13 | 24.53% | 14 | 26.42% | 26 | 49.06% | 0.010 |
|  | III&IV | 66 | 6 | 9.09% | 33 | 50.00% | 27 | 40.91% |  |
| Lymph node metastasis | No | 54 | 12 | 22.22% | 16 | 29.63% | 26 | 48.15% | 0.076 |
|  | Yes | 65 | 7 | 10.77% | 31 | 47.69% | 27 | 41.54% |  |
| Prognosis (Log-rank Mantel-Cox test) | Dead | 73 | 7 | 9.59% | 34 | 46.58% | 32 | 43.84% | 0.020 |
|  | Survival | 46 | 12 | 26.09% | 13 | 28.26% | 21 | 45.65% |  |

| **Supplementary Table 5. The relationships between the model risks and clinicopathological parameters in discovery cohort (GSE53624)** | | | | | | | |
| --- | --- | --- | --- | --- | --- | --- | --- |
| Clinical, epidemiological or pathological feature | | Total (N) | High-risk | Proportion | Low-risk | Proportion | P-value |
|  |  |  |  |  |  |  |  |
|  |  |  |  |  |  |  |  |
| All cases |  | 119 | 30 |  | 89 |  |  |
| Age | <60 | 61 | 17 | 27.87% | 44 | 72.13% | 0.789 |
|  | 60-69 | 45 | 10 | 22.22% | 35 | 77.78% |  |
|  | ≥70 | 13 | 3 | 23.08% | 10 | 76.92% |  |
| Sex | Male | 98 | 25 | 25.51% | 73 | 74.49% | 0.871 |
|  | Female | 21 | 5 | 23.81% | 16 | 76.19% |  |
| Smoking | No | 39 | 9 | 23.08% | 30 | 76.92% | 0.708 |
|  | Yes | 80 | 21 | 26.25% | 59 | 73.75% |  |
| Drinking | No | 45 | 10 | 22.22% | 35 | 77.78% | 0.558 |
|  | Yes | 74 | 20 | 27.03% | 54 | 72.97% |  |
| Location | Upper thoracic | 14 | 5 | 35.71% | 9 | 64.29% | 0.325 |
|  | Middle thoracic | 69 | 14 | 20.29% | 55 | 79.71% |  |
|  | Lower thoracic | 36 | 11 | 30.56% | 25 | 69.44% |  |
| Grade | Grade1 | 23 | 7 | 30.43% | 16 | 69.57% | 0.576 |
|  | Grade2 | 64 | 17 | 26.56% | 47 | 73.44% |  |
|  | Grade3 | 32 | 6 | 18.75% | 26 | 81.25% |  |
| Stage | I&II | 53 | 10 | 18.87% | 43 | 81.13% | 0.153 |
|  | III&IV | 66 | 20 | 30.30% | 46 | 69.70% |  |
| Lymph node metastasis | No | 54 | 12 | 22.22% | 42 | 77.78% | 0.531 |
|  | Yes | 65 | 18 | 27.69% | 47 | 72.31% |  |
| Cluster | Cluster1 | 19 | 0 | 0.00% | 19 | 100.00% | 6.511E-07 |
|  | Cluster2 | 47 | 24 | 51.06% | 23 | 48.94% |  |
|  | Cluster3 | 53 | 6 | 11.32% | 47 | 88.68% |  |
| Prognosis (Log-rank Mantel-Cox test) | Dead | 73 | 27 | 36.99% | 46 | 63.01% | 5.162E-08 |
|  | Survival | 46 | 3 | 6.52% | 43 | 93.48% |  |

| **Supplementary Table 6. The relationships between the model risks and clinicopathological parameters in validation cohort (TCGA)** | | | | | | | |
| --- | --- | --- | --- | --- | --- | --- | --- |
| Clinical, epidemiological or pathological feature | | Total(N) | High-risk | Proportion | Low-risk | Proportion | P-value |
|  |  |  |  |  |  |  |  |
|  |  |  |  |  |  |  |  |
| All cases |  | 95 | 35 |  | 60 |  |  |
| Age | <60 | 57 | 22 | 38.60% | 35 | 61.40% | 0.310 |
|  | 60-69 | 23 | 10 | 43.48% | 13 | 56.52% |  |
|  | ≥70 | 15 | 3 | 20.00% | 12 | 80.00% |  |
| Sex | Male | 81 | 30 | 37.04% | 51 | 62.96% | 0.925 |
|  | Female | 14 | 5 | 35.71% | 9 | 64.29% |  |
| Smoking | No | 59 | 18 | 30.51% | 41 | 69.49% | 0.101 |
|  | Yes | 36 | 17 | 47.22% | 19 | 52.78% |  |
| Drinking | No | 69 | 22 | 31.88% | 47 | 68.12% | 0.103 |
|  | Yes | 26 | 13 | 50.00% | 13 | 50.00% |  |
| Location | Upper thoracic | 45 | 14 | 31.11% | 31 | 68.89% | 0.490 |
|  | Middle thoracic | 44 | 19 | 43.18% | 25 | 56.82% |  |
|  | Lower thoracic | 6 | 2 | 33.33% | 4 | 66.67% |  |
| Grade | Grade1 | 17 | 4 | 23.53% | 13 | 76.47% | 0.115 |
|  | Grade2 | 52 | 24 | 46.15% | 28 | 53.85% |  |
|  | Grade3 | 26 | 7 | 26.92% | 19 | 73.08% |  |
| Stage | I&II | 63 | 18 | 28.57% | 45 | 71.43% | 0.019 |
|  | III&IV | 32 | 17 | 53.13% | 15 | 46.88% |  |
| Lymph node metastasis | No | 55 | 16 | 29.09% | 39 | 70.91% | 0.066 |
|  | Yes | 40 | 19 | 47.50% | 21 | 52.50% |  |
| Prognosis (Log-rank Mantel-Cox test) | Dead | 32 | 16 | 50.00% | 16 | 50.00% | 0.052 |
|  | Survival | 63 | 19 | 30.16% | 44 | 69.84% |  |

| **Supplementary Table 7. The COX regression analysis of ESCC discovery cohort (GSE53624)** | | | | | | | | |
| --- | --- | --- | --- | --- | --- | --- | --- | --- |
| Clinical, epidemiological or pathological feature | Univariate | | | | Multivariate | | | |
|  | P-value | HR | 95%CI | | P-value | HR | 95%CI | |
|  |  |  | Lower limit | Upper limit |  |  | Lower limit | Upper limit |
| Gender(female vs male) | 0.512 | 1.210 | 0.685 | 2.137 |  |  |  |  |
| Age | 0.024 |  |  |  | 0.030 |  |  |  |
| Age(<60-69 vs <60) | 0.462 | 1.207 | 0.731 | 1.991 | 0.547 | 1.169 | 0.703 | 1.945 |
| Age(>=70 vs <60) | 0.006 | 2.583 | 1.306 | 5.108 | 0.008 | 2.524 | 1.267 | 5.026 |
| Location | 0.430 |  |  |  |  |  |  |  |
| Location(middle vs lower) | 0.655 | 0.888 | 0.528 | 1.494 |  |  |  |  |
| Location(upper vs lower) | 0.382 | 1.382 | 0.669 | 2.857 |  |  |  |  |
| Smoking(yes vs no) | 0.535 | 0.859 | 0.532 | 1.388 |  |  |  |  |
| Drinking(yes vs no) | 0.834 | 1.052 | 0.656 | 1.687 |  |  |  |  |
| Stage(III&IV vs I&II) | 0.002 | 2.190 | 1.339 | 3.582 | 0.021 | 1.849 | 1.096 | 3.120 |
| Lymph node metastasis(yes vs no) | 0.002 | 2.159 | 1.319 | 3.534 |  |  |  |  |
| Grade | 0.228 |  |  |  |  |  |  |  |
| Grade(G2 vs G1) | 0.635 | 0.861 | 0.464 | 1.598 |  |  |  |  |
| Grade(G3 vs G1) | 0.363 | 1.362 | 0.700 | 2.651 |  |  |  |  |
| Risk score(high vs low) | 3.008E-07 | 3.617 | 2.212 | 5.914 | 4.100E-05 | 2.955 | 1.761 | 4.961 |

| **Supplementary Table 8. The COX regression analysis of ESCC validation cohort (TCGA)** | | | | | | | | |
| --- | --- | --- | --- | --- | --- | --- | --- | --- |
| Clinical, epidemiological or pathological feature | Univariate | | | | Multivariate | | | |
|  | P-value | HR | 95%CI | | P-value | HR | 95%CI | |
|  |  |  | Lower limit | Upper limit |  |  | Lower limit | Upper limit |
| Gender(female vs male) | 0.026 | 0.190 | 0.044 | 0.819 | 0.038 | 0.195 | 0.041 | 0.916 |
| Age | 0.178 |  |  |  | 0.035 |  |  |  |
| Age(<60-69 vs <60) | 0.972 | 0.985 | 0.413 | 2.349 | 0.404 | 0.675 | 0.268 | 1.699 |
| Age(>=70 vs <60) | 0.074 | 2.402 | 0.918 | 6.287 | 0.023 | 3.232 | 1.177 | 8.873 |
| Location | 0.449 |  |  |  |  |  |  |  |
| Location(middle vs lower) | 0.206 | 1.633 | 0.764 | 3.494 |  |  |  |  |
| Location(upper vs lower) | 0.981 | 0.000 | 0.000 | \ |  |  |  |  |
| Smoking(yes vs no) | 0.731 | 0.876 | 0.413 | 1.858 |  |  |  |  |
| Drinking(yes vs no) | 0.636 | 0.805 | 0.327 | 1.980 |  |  |  |  |
| Stage(III&IV vs I&II) | 0.041 | 2.068 | 1.030 | 4.150 | 0.123 | 1.794 | 0.853 | 3.775 |
| Lymph node metastasis(yes vs no) | 0.136 | 1.699 | 0.846 | 3.413 |  |  |  |  |
| Grade | 0.361 |  |  |  |  |  |  |  |
| Grade(G2 vs G1) | 0.211 | 1.983 | 0.678 | 5.797 |  |  |  |  |
| Grade(G3 vs G1) | 0.658 | 1.321 | 0.386 | 4.525 |  |  |  |  |
| Risk score(high vs low) | 0.056 | 1.971 | 0.982 | 3.955 | 0.011 | 2.670 | 1.250 | 5.704 |
